# Comprehensive artificial intelligence-assisted mental health program for university students in China (CAMPUS): study protocol for a multi-center prospective cohort with planned embedded randomized intervention trials

**DOI:** 10.3389/fpsyg.2026.1877887

**Published:** 2026-10-14

**Authors:** Guang Yang, Rongxun Liu, Chuansheng Wang, Rongxin Zhu, Shisen Qin, Jinnan Yan, Yan Wang, Hongbo Pei, Lijuan Liang, Yingying Su, Shuai Wang, Weidong Wu, Guangjun Ji, Fei Wang, Yange Wei

**Affiliations:** 1 Department of Early Intervention, Mental Health and Artificial Intelligence Research Center, The Second Affiliated Hospital of Henan Medical University, Henan Mental Hospital, Xinxiang, China; 2 School of Public Health, Henan Medical University, Xinxiang, China; 3 International Collaborative Laboratory for Air Pollution Health Effects and Intervention, Henan Medical University, Xinxiang, China; 4 Early Intervention Unit, Department of Psychiatry, Nanjing Brain Hospital, Nanjing Medical University, Nanjing, China; 5 Center for Psychological Development, China Medical University, Shenyang, China; 6 School of Public Health, Lanzhou University, Lanzhou, China; 7 Department of Psychology, The First Affiliated Hospital of Hainan Medical University, Haikou, China; 8 School of Medicine, Southern University of Science and Technology, Shenzhen, China; 9 School of Psychology, Shandong Second Medical University, Weifang, China; 10 Department of Mental Health, School of Public Health, Nanjing Medical University, Nanjing, China; 11 Department of Psychiatry, Yale School of Medicine, New Haven, CT, United States; 12 Department of Psychiatry, Peking University Sixth Hospital, Peking University Institute of Mental Health, NHC Key Laboratory of Mental Health (Peking University), National Clinical Research Center for Mental Disorders (Peking University Sixth Hospital), Beijing, China

**Keywords:** artificial intelligence, digital mental health platform, individualized psychological intervention, multi-center cohort study, multidimensional screening, multi-omics, study protocol, university students

## Abstract

University students face a rising prevalence of depression, anxiety, insomnia, and suicidal ideation that exceeds the capacity of conventional, resource-constrained campus support services. Although artificial intelligence (AI)-assisted screening tools have emerged, many have evaluated selected modalities or isolated prediction tasks, and their integration with prospectively evaluated intervention pathways remains limited. This protocol describes the Comprehensive Artificial Intelligence-Assisted Mental Health Program for University Students in China (CAMPUS), a multi-center research program comprising a five-year prospective cohort, eight planned embedded randomized controlled trials, and exploratory multimodal AI and multi-omics studies across seven Chinese universities. Longitudinal cohort enrolment commenced at Henan Medical University in September 2025 and is planned to expand across seven participating universities. Multidimensional screening integrates psychological self-report (PHQ-9, GAD-7, ISI, BSI-CV, and others), physiological measurements (EEG, MRI, fNIRS, HRV, eye tracking, facial-expression analysis, ERP, voice acoustics), and an exploratory biospecimen component for multi-omics profiling, potentially including genomic, transcriptomic, proteomic, metabolomic, and microbiome analyses, subject to the finalization and approval of layer-specific protocols. Students identified through cohort screening as potentially eligible for an intervention trial will undergo trial-specific eligibility assessment. Those who meet the eligibility criteria and provide trial-specific informed consent will be individually randomized in a 1:1 ratio to the intervention or its comparator within one of eight separate two-arm, multi-center, parallel-group randomized controlled trials. A Transformer-based multimodal AI framework with SHAP-based feature attribution will be developed and internally evaluated for questionnaire-defined risk stratification and exploratory treatment-response prediction. The initial Henan Medical University cohort recruitment and university-student mental-health assessment were approved by the Ethics Review Committee of Henan Medical University (XYLL-20250484), and written informed consent is obtained from participants before enrolment. Each additional participating center will obtain local ethics approval before study activation. Findings will be disseminated through peer-reviewed publications, scientific conferences, and policy briefs, providing evidence that may inform the design and prospective evaluation of youth mental-health programs in other university settings.

**Clinical trial registration:**

The two clinical trials were registered in the Chinese Clinical Trial Registry (ChiCTR). The registration details and URLs are as follows: ChiCTR2500113527: https://www.chictr.org.cn/showproj.html?proj=298271; ChiCTR2500113613: https://www.chictr.org.cn/showproj.html?proj=298268.

## 1 Introduction

University students experience a substantial burden of mental health problems, including depression and suicidal ideation (Auerbach et al., 2016; Rotenstein et al., 2016; Wang et al., 2017). Depression can adversely affect academic productivity among university students (Hysenbegasi et al., 2005). Emotional competence, perceived stress, coping patterns, lifestyle factors, burnout, and environmental exposures have also been associated with student mental health (Ranasinghe et al., 2017; Sun et al., 2017; Piumatti, 2018; van Venrooij et al., 2017; Wang and Liu, 2024). Examining the influence of these various factors and their interactions is critical for understanding the development of mental disorders.

Despite the increasing efforts to identify risk factors associated with mental health issues among university students, existing research remains limited in scope and methodology. Much of the existing literature relies on brief self-report instruments. Measures such as the GAD-7 are useful for assessing anxiety symptoms (Spitzer et al., 2006), but self-report measures alone cannot characterize longitudinal symptom development or determine the incremental contribution of behavioral and physiological information. Reliance on self-report measures alone may limit the ability to identify early-stage or subclinical changes and to characterize their longitudinal development. Furthermore, many existing cohort studies only examine limited variables or short-term outcomes. For example, a longitudinal study conducted in Hebei Province assessed levels of anxiety and depression among 2,748 students at two distinct time points, with a specific emphasis on lifestyle factors (Wang et al., 2025). Although the effects of cohort studies have been examined among university students, their application in managing mental health on the university campus remains limited.

Current mental health assessments in university settings rely heavily on episodic self-report questionnaires, which are susceptible to recall bias and social desirability effects. These single-modality approaches do not capture the broader behavioral and physiological information that may complement self-reported symptoms and support the evaluation of multidimensional screening models. Therefore, there is an urgent need to create a comprehensive mental health screening system on university campuses. Recent advances in behavioral and physiological sensing technologies open new opportunities for objective assessment. Speech-derived acoustic and prosodic features have been investigated for depression and suicide-risk assessment (Cummins et al., 2015). HRV provides a measure of autonomic function, and reduced HRV has been associated with depression (Kemp et al., 2010). Smartphone sensing has also been used to monitor sleep-related patterns among university students in real-world settings (Langholm et al., 2023). fNIRS studies have reported differences in prefrontal activation during emotional processing between depressed individuals and healthy controls (Manelis et al., 2019), while EEG-derived features have been investigated for depression detection (Shen et al., 2022). The reproducibility and incremental screening value of these measures nevertheless require prospective evaluation. Magnetic resonance imaging (MRI) can characterize structural and functional brain features that may be associated with mental-health outcomes; whether such features provide reproducible or clinically useful screening information requires empirical evaluation. Finally, biological samples enable exploratory multi-omics profiling of molecular features associated with mental-health outcomes and may generate candidate signatures for subsequent replication and validation. Nevertheless, fewer studies have integrated psychological, physiological, and biological data into a continuous, comprehensive, and AI-assisted model of mental health evaluation.

The potential value of multidimensional screening also depends on how students identified as being at risk are linked to appropriate assessment and intervention. Mental disorders are common among college students, yet only a minority of affected students receive treatment (Auerbach et al., 2016). Interventions relevant to university students include face-to-face and online individual therapy, group programs, and guided self-help. Internet-based self-help interventions have shown benefits for depression in adolescents and young adults (Ma et al., 2024), although effect sizes and clinical relevance should be interpreted cautiously (Ebert and Baumeister, 2017). Implementation outcomes remain incompletely evaluated: a systematic review of 90 CBT-based digital mental-health intervention studies among college students found that only 19% reported sufficient information to calculate reach and 39% reported uptake (D’Adamo et al., 2023). These findings support prospective evaluation of coordinated pathways linking screening, trial-specific eligibility assessment, and separately evaluated intervention options in university settings. The CAMPUS program therefore brings multidimensional screening and a range of separately evaluated intervention formats together within a coordinated research framework. With this background, our study encompasses a comprehensive array of intervention modalities, including online and face-to-face Interpersonal and Social Rhythm Therapy (IPSRT), Cognitive Behavioral Therapy (CBT), and music intervention. Together, these components create a coordinated research framework linking cohort screening to trial-specific eligibility assessment and separately randomized intervention studies. Whether this framework improves detection, engagement, or mental-health outcomes will be evaluated prospectively. Linking model outputs with physiological and molecular data may generate hypotheses about the biological correlates of mental-health outcomes. Such associations will be evaluated separately from model-level feature attribution and will not be interpreted as evidence of mechanism or causality.

Therefore, this protocol describes the Comprehensive Artificial Intelligence-Assisted Mental Health Program for University Students in China (CAMPUS), a five-year, multicenter research program comprising a prospective longitudinal cohort, eight planned embedded randomized trials, and exploratory multimodal AI and multi-omics analyses. The aims of CAMPUS are to develop and internally evaluate a multidimensional AI-assisted screening framework, to evaluate embedded psychological interventions, and to investigate exploratory molecular correlates of mental-health outcomes as a basis for future mechanistic research.

## 2 Methods and analysis

### 2.1 Study design

This prospective, multicenter, longitudinal cohort study involves collaboration among the study sponsor (oversight), the Mental Health and Artificial Intelligence Research Center (project and data management), Henan Medical University Mental Health Center (site coordination), and site investigators (data collection). The study forms a coordinated, school-based alliance by including participating universities from the north-east (China Medical University), north-west (Lanzhou University), east (Shandong Second Medical University and Nanjing Medical University), south (Southern University of Science and Technology and Hainan Medical University), and central regions (Henan Medical University) of China, spanning seven provinces.

The program comprises three components. Component A is a five-year prospective longitudinal cohort and provides the screening and follow-up framework. Component B comprises eight planned embedded randomized controlled trials. Component C comprises exploratory multimodal AI and multi-omics analyses conducted in eligible cohort or trial subsamples. Table 1 specifies the population, exposure or intervention, outcomes, assessment time points, principal analysis, and inferential designation for each objective and trial. The present article is a master program protocol. It governs the longitudinal cohort, shared screening and safety procedures, common assessment framework, data governance, and the overarching design of the planned embedded trials. It is not the definitive trial-specific protocol for any individual intervention trial. Before activation and the first participant randomization of an embedded trial, its final eligibility criteria, comparator, intervention format and dose, provider requirements, fidelity procedures, intervention manual, statistical analysis plan, ethics approval, and registry record will be prospectively finalized and documented. Detailed specifications of the AI tasks, computational architecture, embedded trial design, assessment schedule, multi-omics analysis plan, component-specific safety procedures, and intervention-planning matrix are provided in Supplementary Tables 1–7, respectively.

**Table 1:** Pre-specified objectives, outcomes, and analysis strategy within the CAMPUS program.

Objective	Population	Exposure/Intervention	Comparator	Primary outcome	Secondary outcomes	Assessment time point	Principal analysis	Designation
A1. Estimate the five-year trajectory of depressive symptoms	Undergraduate and postgraduate students recruited from the seven participating universities and followed for up to 5 years	—	—	Repeated PHQ-9 score across baseline and nine semester follow-up waves	Repeated GAD-7, ISI, PSS-10, BSI-CV, SHAPS, TEPS and MPATS scores; incident suicidal ideation	Baseline and each semester follow-ups	Weighted pseudo-likelihood mixed-effects model with semester wave, university, sex, and academic stage as fixed effects; participant-level random intercept and time slope; primary-sampling-unit random intercept; design-based sandwich variance accounting for sampling strata and primary sampling units	Primary pre-specified longitudinal cohort analysis (estimation-focused); secondary outcomes and effect-modification analyses are exploratory
A2. Examine multimodal predictors of subsequent mental health problems	Cohort participants meeting the data requirements for the relevant prediction task; participants with baseline PHQ-9 <10 for the incident depressive-symptom task	Pre-specified baseline questionnaire, behavioral, physiological and neuro-functional features	—	Time to the first follow-up PHQ-9 score ≥10	Longitudinal PHQ-9 slope and pre-specified depressive-symptom trajectory parameters	Baseline and each semester follow-ups	Discrete-time survival modeling for incident depressive symptoms and pre-specified benchmark models for trajectory parameters, with participant-level internal validation	Exploratory prediction and hypothesis generation
B1. CBT: evaluate the intervention effect on its target outcome	Students with depressive symptoms defined by a PHQ-9 score of 5–14 and no current psychological treatment	CBT	Trial-specific control condition to be finalized and prospectively specified before trial activation.	Between-group difference in PHQ-9 change from baseline to the scheduled post-intervention assessment	GAD-7, ISI, PSS-10 and BSI-CV	Baseline; within 7 days after the scheduled final intervention session (primary time point); 3-month follow-up	ITT mixed-effects model including treatment, time, treatment × time, baseline primary-outcome score and site as fixed effects and participant as a random intercept	Confirmatory for the trial-specific primary outcome at post-intervention; exploratory for all other analyses
B2. IPSRT: evaluate the intervention effect on its target outcome	Students with subclinical insomnia or circadian-rhythm complaints (ISI 8–14)	IPSRT	Trial-specific control condition to be finalized and prospectively specified before trial activation	Between-group difference in ISI change from baseline to the scheduled post-intervention assessment	PHQ-9, GAD-7, PSS-10 and BSI-CV	Baseline; within 7 days after the scheduled final intervention session (primary time point); 3-month follow-up	ITT mixed-effects model including treatment, time, treatment × time, baseline primary-outcome score and site as fixed effects and participant as a random intercept; primary contrast at post-intervention	Confirmatory for the trial-specific primary outcome at post-intervention; exploratory for all other analyses
B3. Physical exercise: evaluate the intervention effect on its target outcome	Students with depressive symptoms defined by a PHQ-9 score of 5–14 who can safely undertake the planned exercise program	Structured physical exercise	Trial-specific control condition to be finalized and prospectively specified before trial activation	Between-group difference in PHQ-9 change from baseline to the scheduled post-intervention assessment	GAD-7, ISI, PSS-10 and BSI-CV	Baseline; within 7 days after the scheduled final intervention session (primary time point); 3-month follow-up	ITT mixed-effects model including treatment, time, treatment × time, baseline primary-outcome score and site as fixed effects and participant as a random intercept	Confirmatory for the trial-specific primary outcome at post-intervention; exploratory for all other analyses
B4. Planned personalized music intervention: evaluate the intervention effect on its target outcome	Students with subclinical anxiety symptoms (GAD-7: 5–14)	Music intervention	Trial-specific control condition to be finalized and prospectively specified before trial activation	Between-group difference in GAD-7 change from baseline to the scheduled post-intervention assessment	PHQ-9, ISI, PSS-10 and BSI-CV	Baseline; within 7 days after the scheduled final intervention session (primary time point); 3-month follow-up	ITT mixed-effects model including treatment, time, treatment × time, baseline primary-outcome score and site as fixed effects and participant as a random intercept	Confirmatory for the trial-specific primary outcome at post-intervention; exploratory for all other analyses
B5. Biofeedback: evaluate the intervention effect on its target outcome	Students with moderate perceived stress (PSS-10: 14–26) and no contraindication to the biofeedback procedure	Biofeedback	Trial-specific control condition to be finalized and prospectively specified before trial activation	Between-group difference in PSS-10 change from baseline to the scheduled post-intervention assessment	PHQ-9, GAD-7, ISI and BSI-CV	Baseline; within 7 days after the scheduled final intervention session (primary time point); 3-month follow-up	ITT mixed-effects model including treatment, time, treatment × time, baseline primary-outcome score and site as fixed effects and participant as a random intercept	Confirmatory for the trial-specific primary outcome at post-intervention; exploratory for all other analyses
B6. Virtual reality therapy: evaluate the intervention effect on its target outcome	Students with subclinical depressive symptoms (PHQ-9: 5–14) and no contraindication to VR exposure	VR therapy	Trial-specific control condition to be finalized and prospectively specified before trial activation	Between-group difference in PHQ-9 change from baseline to the scheduled post-intervention assessment	GAD-7, ISI, PSS-10 and BSI-CV	Baseline; within 7 days after the scheduled final intervention session (primary time point); 3-month follow-up	ITT mixed-effects model including treatment, time, treatment × time, baseline primary-outcome score and site as fixed effects and participant as a random intercept	Confirmatory for the trial-specific primary outcome at post-intervention; exploratory for all other analyses
B7. Manualized expressive-arts intervention: evaluate the intervention effect on its target outcome	Students with subclinical anxiety symptoms (GAD-7: 5–14)	Expressive art intervention	Trial-specific control condition to be finalized and prospectively specified before trial activation	Between-group difference in GAD-7 change from baseline to the scheduled post-intervention assessment	PHQ-9, ISI, PSS-10 and BSI-CV	Baseline; within 7 days after the scheduled final intervention session (primary time point); 3-month follow-up	ITT mixed-effects model including treatment, time, treatment × time, baseline primary-outcome score and site as fixed effects and participant as a random intercept	Confirmatory for the trial-specific primary outcome at post-intervention; exploratory for all other analyses
B8. Five-sensory stress-regulation intervention: evaluate the intervention effect on perceived stress	Students with a PSS-10 score of 14–26 at the trial-specific eligibility assessment and no trial-specific contraindication	Manualized five-sensory stress-regulation intervention	Trial-specific control condition to be finalized and prospectively specified before trial activation	Adjusted between-group difference in PSS-10 change from baseline to the scheduled post-intervention assessment	PHQ-9, GAD-7 and ISI; BSI-CV as a safety outcome	Baseline; within 7 days after the scheduled final intervention session (primary time point); 3-month follow-up	ITT mixed-effects model including treatment, time, treatment × time, baseline PSS-10 score and site as fixed effects and participant as a random intercept	Confirmatory for the pre-specified PSS-10 outcome at the primary post-intervention time point; exploratory for secondary outcomes, maintenance, subgroup, and other analyses
C1. Develop and internally evaluate questionnaire-defined screening and risk-stratification models	Cohort participants with the predictors and questionnaire-defined reference outcome required for each task	Questionnaire, behavioral, physiological and neuro-functional features	Unimodal models, regularized regression, and gradient-boosting models fitted to concatenated preprocessed features	Questionnaire-defined depressive-symptom status at the same wave (primary threshold PHQ-9 ≥10)	Questionnaire-defined anxiety, insomnia and suicidal-ideation outcomes	Same-wave predictors and reference outcomes at eligible semester assessments	Nested participant-level internal validation, temporal validation and leave-one-site-out validation	Exploratory; no diagnostic or independent external-validation claim
C2. Examine whether early multimodal indicators predict trial-specific treatment outcomes	Component B participants with the early-phase predictors and reference outcomes required for the relevant model	Early treatment-phase behavioral, physiological and self-report indicators	Pre-specified regularized regression, gradient-boosting, and unimodal benchmark models, as applicable	Continuous change from baseline in the trial-specific primary outcome measured within 7 days after the scheduled final intervention session	Clinically meaningful improvement where a validated threshold is pre-specified; symptom status at 3 months	Early treatment phase; post-intervention and 3-month follow-up	Pre-specified benchmark and longitudinal prediction models with participant-level and site-aware validation	Exploratory prediction analysis
C3. Examine molecular correlates of mental-health outcomes and treatment response	Participants in the bio-specimen sub-cohort with the omics layers and outcomes required for each analysis	Available assay-quality molecular features from omics layers activated under pre-specified layer-specific protocols, potentially including genomic, DNA-methylation, transcriptomic, proteomic, metabolomic, lipidomic, and microbiome data.	-	Feature-level associations with depressive-symptom severity at baseline and over follow-up	Associations with incident depressive symptoms and trial-specific outcomes; multi-omics latent factors; pathway and gene-set enrichment	Baseline and available follow-up bio-specimens; semester and trial assessments where applicable	Layer-specific association models with FDR correction; MOFA+ for unsupervised integration; pre-specified enrichment and validation analyses	Exploratory and hypothesis-generating; no mechanistic or causal claims

The Component B entries describe planned trial questions and provisional core design features within the CAMPUS master program protocol. They do not replace the final trial-specific protocol, statistical analysis plan, intervention manual, ethics approval, or registry record required before activation of an individual trial. Each embedded trial is a separate confirmatory comparison. No participant is randomized among the eight interventions, and algorithmic recommendations do not determine trial allocation. AI, artificial intelligence; BSI-CV, Beck Scale for Suicide Ideation; FDR, false-discovery rate; GAD-7, Generalized Anxiety Disorder-7; ISI, Insomnia Severity Index; ITT, intention-to-treat; MOFA+, multi-omics factor analysis+; MPATS, Mobile Phone Addiction Tendency Scale; PHQ-9, Patient Health Questionnaire-9; PSS-10, 10-Item Perceived Stress Scale; SHAPS, Snaith–Hamilton Pleasure Scale; TEPS, Temporal Experience of Pleasure Scale.

#### 2.1.1 Component A: multicenter prospective longitudinal cohort

Students will be recruited from the seven participating universities using stratified multistage probability sampling and assessed once per semester over 5 years. The primary cohort objective (A1) is to estimate the longitudinal trajectory of depressive symptoms. Analyses of anxiety, insomnia, stress, suicidal ideation, anhedonia, mobile-phone addiction, and incident suicidal ideation are secondary estimates and will be interpreted as exploratory. Objective A2 examines whether pre-specified baseline questionnaire, behavioral, physiological, and neuro-functional characteristics predict the first subsequent PHQ-9 score ≥10 among participants with baseline PHQ-9 <10 and pre-specified longitudinal depressive-symptom trajectory parameters during subsequent semester follow-up waves. Because these predictor analyses involve model development and multiple candidate features, they are designated exploratory.

#### 2.1.2 Component B: embedded randomized intervention trials

Students identified through cohort screening as potentially eligible for an intervention trial may undergo trial-specific eligibility assessment. The eight interventions will be evaluated in eight separate two-arm, multicenter, parallel-group randomized controlled trials using individual 1:1 randomization. Each trial will have its own eligibility criteria, intervention protocol, trial-specific comparator, primary outcome, and assessment schedule. For each trial that has not yet been activated, the comparator and its rationale will be finalized and prospectively documented before trial activation and the first participant randomization. Confirmatory inference will be restricted to the pre-specified primary outcome and primary time point of each trial. Secondary outcomes, subgroup analyses, and pooled analyses across trials will be considered exploratory. All eight embedded trials remain in the planning stage. The descriptions in this master protocol provide a program-level framework and do not constitute final trial-specific protocols, ethics approvals, intervention manuals, statistical analysis plans, or registry records. Each trial will be activated only after its trial-specific protocol, ethics approval, intervention manual, statistical analysis plan, and prospective public registration have been completed.

#### 2.1.3 Component C: exploratory multimodal and multi-omics studies

Component C will address three exploratory objectives. C1 concerns the development and internal evaluation of models for questionnaire-defined mental health screening and risk stratification. C2 examines whether pre-specified baseline and early-treatment behavioral, physiological, and self-report indicators predict the continuous trial-specific primary outcome at the scheduled post-intervention assessment. C3 investigates molecular features and multi-omics patterns associated with mental health outcomes and intervention response. These analyses are intended to evaluate prediction performance, identify reproducible associations, and generate hypotheses for subsequent research.

### 2.2 Participants and recruitment

Participation is voluntary, and participants may withdraw consent for continued participation at any time. The inclusion criteria for the longitudinal cohort are: (1) age 18–30 years, inclusive; (2) current enrolment at a participating university; (3) willingness to complete the minimum core cohort assessment, defined as the self-report questionnaire battery applicable to the participant’s academic stage, independently or with reasonable accommodation; and (4) provision of valid written informed consent. Exclusion criteria are: (1) a prior clinical diagnosis of major depressive disorder or generalized anxiety disorder; or (2) an acute cognitive, medical, or psychiatric state that prevents the individual from understanding the study information, providing valid informed consent, or safely completing the minimum core cohort assessment despite reasonable accommodation. When such a condition is temporary, enrolment may be deferred and reconsidered after the condition has resolved. Inability or unwillingness to complete an individual optional component, including voice or facial-expression recording, EEG, ERP, HRV, eye tracking, fNIRS, MRI, or biospecimen collection, will not exclude the participant from the longitudinal cohort. Each component will be subject to component-specific consent or consent options, safety screening, participant tolerance, and technical feasibility. Participants who decline or cannot safely complete one component may continue with all other components that are safe and acceptable. Participant refusal, safety contraindication, temporary deferral, technical failure, inability to obtain usable data, and quality-control exclusion will be documented separately. Reasonable accommodations may include accessible electronic or large-print materials, screen-reader compatibility, verbatim staff administration with the participant’s own responses recorded, additional time and breaks, shorter or divided sessions, flexible scheduling, accessible testing locations, positioning support, and modality-specific adjustments that do not compromise safety or measurement validity.

Within each center and annual recruitment cycle, eligible participants from the core cohort will be stratified by sex and academic stage. Within each stratum, participants will be assigned a computer-generated random invitation order. Invitations will be issued in that order until the center-specific annual target is reached or the eligible sampling frame is exhausted. If a selected participant declines, does not attend, does not provide component-specific consent, has a safety contraindication, or cannot complete the scheduled assessment, the next eligible participant in the same stratum will be invited. Where a stratum is exhausted, any remaining places may be allocated using the randomized invitation order in the remaining strata, with the deviation documented. Additional invitations may continue in the pre-specified random order while capacity remains. Participants will not be selected or replaced on the basis of symptom outcomes, physiological findings, or model results.

For each participant considered for the multimodal component, the study will record the sampling stratum, invitation status, response, component-specific consent, attendance, safety contraindications, non-participation, completed modalities, technical failures, quality-control eligibility, and reasons for unavailable data. Analyses involving multimodal data will target participants selected for the relevant modality-availability block and with usable data for the required measures. Characteristics of invited, enrolled, and analytically eligible multimodal participants will be reported and compared with those of the relevant core-cohort sampling frame using available baseline variables. Stratum-specific selection fractions and modality-specific completion rates will also be reported.

The seven universities were selected through established research collaborations. The target population for cohort inference comprises eligible students aged 18–30 years who are enrolled at the seven participating universities. Within each participating university, the current student enrolment register will provide the sampling frame. Students will be stratified jointly by academic year and major discipline. Within each university, the operational recruitment target of approximately 6,000 participants will be allocated across the academic-year-by-major-discipline strata in proportion to official enrolment counts. Classes or administrative groups within each stratum will then be randomly selected as primary sampling units, after which individual students will be randomly selected from the selected units using computer-generated lists. If operational constraints result in disproportionate sampling or deviations from the planned stratum allocation, the realized selection probabilities will be reflected in the sampling weights. The numbers included in the sampling frame, randomly selected, contacted, screened, eligible, consenting, and enrolled will be recorded by university and, where cell sizes permit, by academic-year and major-discipline stratum. Site-specific recruitment totals, participation rates with clearly defined denominators, and material deviations from the planned allocation will be reported. The recruitment and consent procedures at Henan Medical University were approved by the Ethics Review Committee of Henan Medical University (approval no. XYLL-20250484).

#### 2.2.1 Sample size and statistical power

Longitudinal cohort (Component A). The prospective cohort will aim to enroll approximately 42,000 students at baseline, with an operational target of approximately 6,000 participants from each of the participating universities. The final contribution of an individual university may vary modestly according to its eligible student population and recruitment capacity. Site-specific recruitment totals and any material deviations from the operational targets will be reported.

For illustrative precision calculations, a PHQ-9 standard deviation of 4.0 is assumed. With 42,000 participants, the approximate 95% confidence-interval half-width for a wave-specific mean is 0.04 points under simple random sampling and 0.05 points under an illustrative design effect of 2.0. Assuming 15% annual attrition, approximately 18,600 participants would remain after 5 years, corresponding to a half-width of approximately 0.08 points under the same standard-deviation and design-effect assumptions. These are planning benchmarks rather than observed study results or a power calculation for a single longitudinal contrast. Among the approximately 35,700–37,800 participants expected to have baseline PHQ-9 scores below 10, cumulative-incidence scenarios of 5–15% would correspond to approximately 1,800–5,700 incident events before allowing for incomplete follow-up. These figures are planning scenarios rather than assumed incidence estimates. The A1 analysis is a primary pre-specified, estimation-focused longitudinal cohort analysis. No single confirmatory null hypothesis, effect-size target, or hypothesis-testing decision rule is pre-specified.

Embedded intervention trials (Component B). Each trial will use an individually randomized, two-arm design. In an unclustered comparison, 130 evaluable participants per arm would provide 80% power to detect a standardized between-group difference of *d* = 0.35 at a two-sided *α* of 0.05. For group-delivered interventions, the calculation allows for partial nesting within the intervention arm, assuming a mean group size of eight and an intra-class correlation coefficient of 0.08, increasing the requirement to approximately 168 evaluable participants per arm. Sensitivity calculations considered intervention-group sizes of 6–10 participants and intra-class correlation coefficients of 0.02–0.10. Allowing for 20% loss of primary-outcome data, each trial will recruit 210 participants per arm (420 per trial; 3,360 across eight trials). Each trial will have one pre-specified primary outcome and one primary post-intervention assessment. The primary intention-to-treat analysis will account for repeated measurements, study site, and intervention-group or provider clustering where applicable. If the final comparator introduces clustering in both trial arms, the sample-size requirement will be reassessed before trial activation and the first participant randomization.

Multimodal subsample (Components A and C). Within each center and annual recruitment cycle, up to 400 participants will be selected from the eligible core cohort using the pre-specified stratified random invitation procedure, yielding up to 14,000 unique participants across the seven centers over 5 years. All participants will be scheduled for the core multimodal battery, including EEG, ERP, HRV, fNIRS, MRI, eye tracking, voice recording, and facial-expression recording, subject to consent, safety screening, and technical feasibility. MRI will not have a separate recruitment target. Sensitivity calculations indicate that approximately 175, 98, and 63 participants per group would be required to detect standardized differences of *d* = 0.30, 0.40, and 0.50, respectively, with 80% power and a two-sided *α* of 0.05. These figures are benchmarks rather than formal power calculations for the prediction models.

Multi-omics sub-cohort (Component C). The initial multi-omics target is 3,000 participants drawn from the multimodal subsample. Approximately 1,300 evaluable participants provide 80% power to detect a correlation of *r* = 0.15 using a two-sided significance threshold of 5 × 10^−6^. This threshold represents an illustrative Bonferroni correction for 10,000 feature-level tests. The calculation is a sensitivity benchmark for a single-feature association and is not a universal power calculation for all omics layers or integrated multi-omics models. The larger recruitment target allows for biospecimen loss, assay failure, incomplete omics layers, covariate adjustment, and internal validation.

#### 2.2.2 Primary and secondary outcomes

The pre-specified outcomes are summarized in Table 2. For Component A, the primary outcome is the population-average trajectory of repeated depressive scores from baseline through follow-up waves, estimated using the pre-specified mixed-effects model rather than separate wave-specific primary endpoints. Repeated GAD-7, ISI, PSS-10, BSI-CV, SHAPS, TEPS, and MPATS scores are secondary outcomes. Incident suicidal ideation is defined as the first follow-up assessment with any positive BSI-CV response among participants without a positive response at baseline. This is an operational questionnaire-based outcome rather than a clinical risk cutoff.

**Table 2:** Pre-specified clinical outcomes of the CAMPUS program.

Outcome domain	Instrument	Operational definition	Range/direction	Threshold	Timing	Pre-specified role	Clinically meaningful change
Depressive symptoms	PHQ-9 (Kroenke et al., 2001)	Total score (9 items)	0–27; higher = worse	≥5 mild; ≥10 moderate-to-severe/screening-positive; <5 remission	Baseline and nine semester follow-up waves in Component A; trial baseline, within 7 days after the scheduled final intervention session, and 3-month follow-up in Component B	Primary cohort outcome (A1); primary outcome in B1 CBT, B3 exercise and B6 VR; secondary in B2/B4/B5/B7/B8; C1 depressive screening reference	A reduction of at least 5 points, used for clinical interpretation and exploratory responder analyses (Löwe et al., 2004)
Anxiety symptoms	GAD-7 (Spitzer et al., 2006)	Total score (7 items)	0–21; higher = worse	≥5 mild; ≥10 moderate	Baseline and nine semester follow-up waves in Component A; trial baseline, within 7 days after the scheduled final intervention session, and 3-month follow-up in Component B	Primary outcome in B4 music and B7 expressive art; secondary cohort outcome and secondary in other trials; C1 anxiety-screening reference	A reduction of at least 4 points, used for clinical interpretation and exploratory responder analyses (Toussaint et al., 2020)
Insomnia symptoms	ISI (Bastien et al., 2001)	Total score (7 items)	0–28; higher = worse	0–7, no clinically significant insomnia; 8–14, subthreshold insomnia; 15–21, moderate insomnia; 22–28, severe insomnia	Baseline and nine semester follow-up waves in Component A; trial baseline, within 7 days after the scheduled final intervention session, and 3-month follow-up in Component B	Primary outcome in B2 IPSRT; secondary longitudinal outcome in Component A and secondary outcome in the other embedded trials; questionnaire-defined reference outcome in the exploratory C1 insomnia-screening task	A reduction of at least 6 points, used for clinical interpretation and exploratory responder analyses (Yang et al., 2009)
Perceived stress	PSS-10 (Cohen et al., 1983)	Total score (10 items)	0–40; higher = worse	No validated diagnostic cut-off pre-specified; scores of 14–26 are used only as a study-defined eligibility range for B5 and B8	Baseline and nine semester follow-up waves in Component A; trial baseline, within 7 days after the scheduled final intervention session, and 3-month follow-up in Component B	Primary outcome in B5 biofeedback and B8 five-sensory stress-regulation trials; secondary longitudinal outcome in Component A and secondary outcome in the other embedded trials	No universally accepted minimal clinically important difference pre-specified; analyzed as a continuous outcome
Suicidal ideation	BSI-CV (Li et al., 2011)	Total score across 19 items; items 1–5 assess suicidal ideation and items 6–19 assess suicidal tendency	0–38; higher scores indicate more severe suicidal ideation and suicidal tendency	Any positive response triggers the safety protocol; modeling threshold defined separately	Baseline and nine semester follow-up waves in Component A; trial baseline, within 7 days after the scheduled final intervention session, and 3-month follow-up in Component B; real-time safety monitoring at every administration	Secondary safety outcome in the cohort and all trials; exploratory C1 questionnaire-defined classification reference	Not established; not used as a confirmatory efficacy outcome
Anhedonia	SHAPS (Snaith et al., 1995; Liu et al., 2012)	Total score (14 items)	0–14; higher = worse	No diagnostic threshold is pre-specified; a score of ≥3 may be reported only as a descriptive operational classification	Baseline and nine semester follow-up waves in Component A	Secondary/exploratory outcome	Not established for this protocol
Anticipatory and consummatory pleasure	Chinese 20-item TEPS (Chan et al., 2012)	Total score (20 items)	20–120; higher = better	Not applicable	Baseline and nine semester follow-up waves in Component A	Secondary/exploratory outcome	Not established for this protocol
Mobile-phone addiction	MPATS (Xiong et al., 2012)	Total score (16 items)	16–80; higher = worse	No validated diagnostic cut-off pre-specified; analyzed as a continuous score	Baseline and nine semester follow-up waves in Component A	Secondary/exploratory outcome	Not established for this protocol
Childhood maltreatment	CTQ-SF (Bernstein et al., 2003; He et al., 2019)	Total score based on 25 clinical items and five subscale scores; three minimization/denial items will be considered separately	25–125 total; higher = worse	No diagnostic threshold is pre-specified; total and subscale scores will be analyzed continuously. Any descriptive categorization will be labeled as operational.	Baseline only	Covariate and exploratory moderator; not an outcome	Not applicable

BSI-CV, Chinese version of the Beck Scale for Suicide Ideation; CTQ, Childhood Trauma Questionnaire; GAD-7, Generalized Anxiety Disorder-7; ISI, Insomnia Severity Index; MCID, minimal clinically important difference; MPATS, Mobile Phone Addiction Tendency Scale; PHQ-9, Patient Health Questionnaire-9; PSS-10, 10-Item Perceived Stress Scale; SHAPS, Snaith–Hamilton Pleasure Scale; TEPS, Temporal Experience of Pleasure Scale. Clinically meaningful change thresholds are used only for interpretation and exploratory responder analyses and do not replace the continuous primary analyses. The PSS-10 range of 14–26 is a study-defined eligibility criterion for B5 and B8 and is not a validated diagnostic cut-off.

Each embedded trial has one trial-specific primary outcome: PHQ-9 for B1, B3, and B6; ISI for B2; GAD-7 for B4 and B7; and PSS-10 for B5 and B8. The primary endpoint is the adjusted between-group difference in change from baseline to the assessment completed within 7 days after the scheduled final intervention session, irrespective of intervention adherence. Three-month maintenance outcomes, secondary outcomes, and subgroup analyses are exploratory. Physiological and neuro-functional measures are candidate predictors in A2, C1, and C2, while multi-omics analyses under C3 are exploratory.

### 2.3 Procedure

#### 2.3.1 Study procedures and data collection

A centralized digital platform (AI Muxin), developed by the research team, will be used to coordinate all procedures across the seven universities. Investigators at the coordinating center will oversee platform operations, site activation, and protocol adherence. All research personnel will undergo standardized training to ensure consistency in psychological assessment, biospecimen management, and data collection procedures. Following online eligibility screening, eligible participants will be invited to an in-person structured assessment session. This session includes: (1) completing digital self-report psychological assessment questionnaires via the AI Muxin application; and (2) undergoing physiological measurements at designated research sites. The biospecimen component has not yet been activated. Blood, urine, stool, and saliva are candidate biological matrices, but the collection schedule and matrix-specific procedures will be finalized before activation. No biospecimen collection will begin until the relevant standard operating procedures, consent materials, and required ethics amendments have been approved. Students identified through cohort screening as potentially eligible for an embedded trial may be invited to undergo trial-specific eligibility assessment. Cohort screening or AI-generated model outputs will not automatically enroll a participant in an intervention or determine trial selection or randomized allocation. Participation in an embedded trial will require confirmation of the trial-specific eligibility criteria, trial-specific informed consent, and completion of the baseline assessment before randomization within the selected trial. These programs will be administered by trained personnel in accordance with standardized protocols. A centralized dashboard system will be used to monitor data quality, ensuring data accuracy and comparability across different sites (Figure 1).

**Figure 1:**
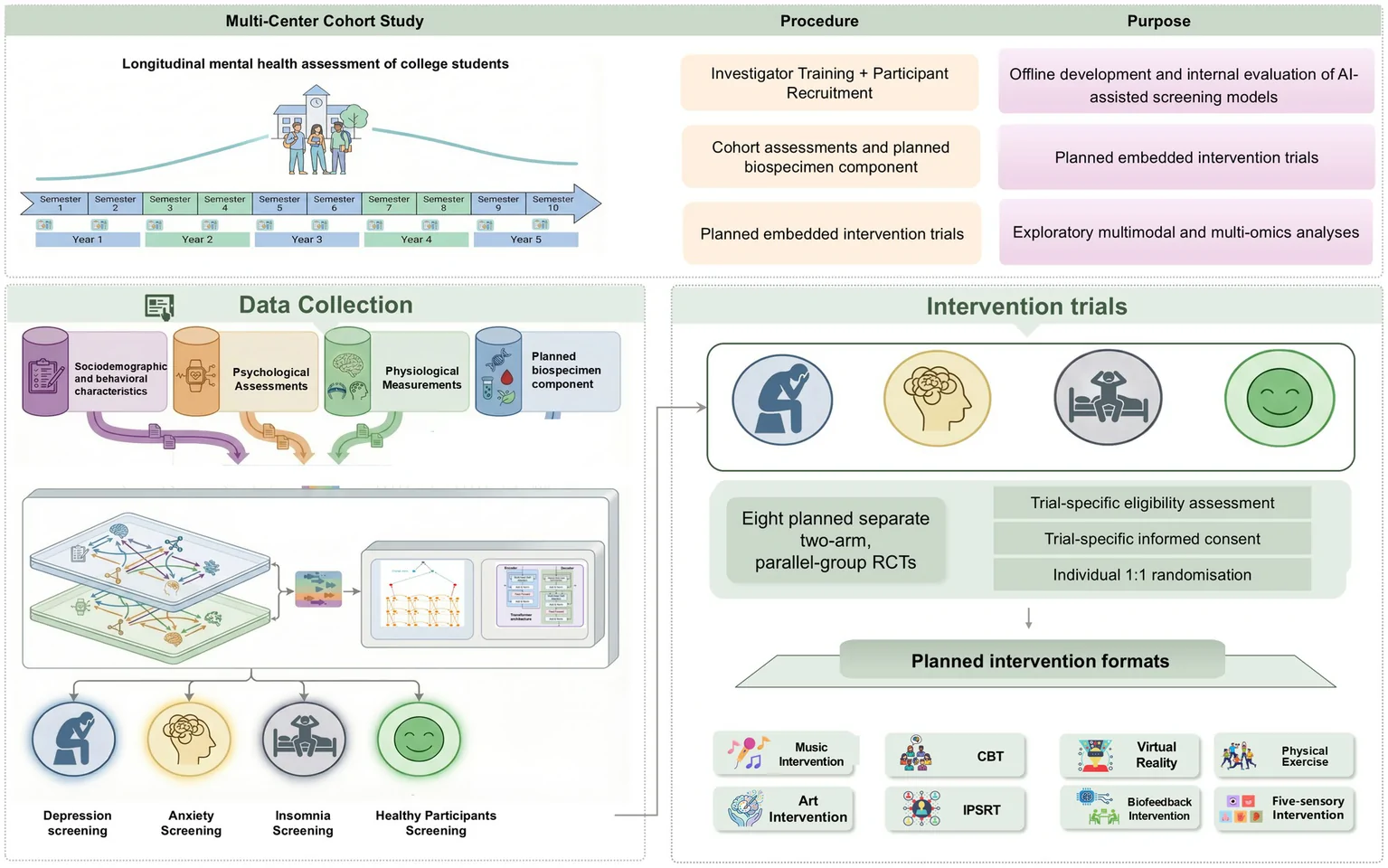
CAMPUS study design, assessment framework, and embedded intervention trials. AI, artificial intelligence; CBT, cognitive behavioral therapy; IPSRT, interpersonal and social rhythm therapy; RCTs, randomized controlled trials.

#### 2.3.2 Safety monitoring and risk management

Suicidal ideation will be assessed at every scheduled questionnaire wave and trial assessment using the BSI-CV. Any positively endorsed BSI-CV item will generate an immediate electronic safety alert routed to trained site staff and the designated site safety officer. Each alert will receive prompt human review to determine whether urgent same-day contact and assessment are required. The BSI-CV is an initial questionnaire-based safety screen and does not determine clinical risk level or disposition. Risk level and disposition will be determined by an appropriately qualified mental health professional using a structured assessment of current ideation, intent, plan, access to means, preparatory behavior, previous suicidal behavior, relevant risk and protective factors, and the participant’s ability to remain safe. AI models will be developed and evaluated offline; their outputs will not be used for real-time safety management, clinical risk assignment, or disposition and will not replace or delay professional assessment or emergency action. A three-level response pathway is pre-specified.

*Level 1*: a positive screen that, following prompt human contact, is assessed as non-acute, with no current intent, feasible plan, or preparatory behavior. Trained study staff will contact the participant within 24 h. A licensed mental health professional will complete a structured suicide safety assessment within 48 h, determine the appropriate disposition, jointly develop or review a personal safety plan, provide local crisis and mental health resources, and arrange follow-up according to clinical need.

*Level 2*: active suicidal ideation with intent, a feasible plan, preparatory behavior, or inability to maintain safety. An immediate same-day assessment by a psychiatrist, licensed clinical psychologist, or other appropriately qualified mental health professional will be arranged. The participant will remain in contact with study or clinical personnel until an appropriate disposition has been established. Where urgent specialist care is required, the participant will be referred to the designated local psychiatric service or emergency department through the site-specific referral pathway.

*Level 3*: imminent risk. If the professional assessment identifies current intent with a feasible plan, ongoing preparatory behavior, a recent suicide attempt, or another indication of imminent danger, the participant will not be left alone. Emergency services will be contacted, and the participant will be transferred to the designated local psychiatric emergency service or emergency department. An emergency contact or another support person identified by the participant will be notified where clinically necessary and consistent with applicable law and institutional policy. Access to potentially lethal means will be addressed as part of the immediate safety response where feasible.

During longitudinal and intervention-trial follow-up, any newly endorsed BSI-CV item will reactivate the suicide-safety pathway. A new PHQ-9 score of 15 or above or an increase of at least 5 points will trigger prompt clinical review, including reassessment of suicidal ideation, but will not by itself determine the suicide-risk level. Escalation will be based on the professional assessment. Before activation, each participating site will establish a written local referral and emergency-response pathway specifying the responsible clinical personnel, designated psychiatric service or emergency department, emergency contact procedures, and arrangements for out-of-hours escalation.

All safety alerts, professional assessments, safety plans, referrals, emergency transfers, suicide attempts, psychiatric hospitalizations, and other relevant adverse events will be documented in the study database. Serious safety events will be reported to the site principal investigator, the coordinating-center safety oversight committee, and the relevant ethics committee within the applicable institutional reporting timelines. A designated qualified site safety officer will hold clinical responsibility for implementation of the pathway at each university. The coordinating-center safety oversight committee will review aggregate safety data and protocol compliance at pre-specified intervals. Participants in all arms of the embedded trials will receive the same safety monitoring and escalation procedures.

#### 2.3.3 Assessment schedule

Questionnaire and platform-based assessments, comprising self-report questionnaires, voice recording, and facial-expression recording, will be administered to the full cohort at baseline and at each of the nine scheduled semester follow-up waves during the five-year study period. The multimodal subsample will be recruited on a rolling basis using the stratified random invitation procedure described in Section 2.2, with an operational target of up to 400 new participants per center per year. Participants enrolled in the multimodal subsample will be scheduled to complete the core physiological and neuro-functional battery, comprising EEG, ERP, HRV, eye tracking, fNIRS, and MRI, at enrolment and annually thereafter until the end of the study period. Participants recruited during the first annual recruitment cycle may therefore contribute up to four annual follow-up assessments, whereas participants recruited in later cycles will contribute fewer follow-up assessments. MRI will follow the same schedule as the other core modalities, subject to pre-specified safety contraindications. The biospecimen component has not yet been activated. Before collection begins, the enrolment and follow-up schedule for each biological matrix will be specified in a matrix-specific standard operating procedure and approved through the applicable ethics and protocol-amendment process. Multi-omics assays will initially target approximately 3,000 participants with assay-eligible specimens, and repeat assays will depend on the pre-specified omics question and the availability of suitable follow-up specimens. The complete schedule, including the relevant population, participating sites, and planned number of observations for each measure, is provided in Supplementary Table 4. Completion of an individual modality will be subject to component-specific consent, safety screening, participant tolerance, and technical feasibility. Declining or being unable to complete one modality will not affect continued participation in the cohort or eligibility for the remaining assessments.

### 2.4 Data collection

#### 2.4.1 Sociodemographic and behavioral characteristics

At baseline, sociodemographic information will be collected, including age, sex, ethnicity, only-child status, and household registration type. Lifestyle behaviors such as smoking, alcohol use, diet, and physical activity will be assessed through structured questionnaires. These variables will serve as key covariates, moderators, or mediators in stratified and adjusted analyses.

#### 2.4.2 Psychological assessments

All participants will complete a set of self-report psychological instruments. Undergraduates will be assessed using the Patient Health Questionnaire-9 (PHQ-9), Generalized Anxiety Disorder-7 (GAD-7), Insomnia Severity Index (ISI), 10-Item Perceived Stress Scale (PSS-10), Chinese version of the Beck Scale for Suicide Ideation (BSI-CV), Snaith-Hamilton Pleasure Scale (SHAPS; Snaith et al., 1995; Liu et al., 2012), Chinese 20-item Temporal Experience of Pleasure Scale (TEPS; Chan et al., 2012), Mobile Phone Addiction Tendency Scale (MPATS; Xiong et al., 2012), and Childhood Trauma Questionnaire-Short Form (CTQ-SF; Bernstein et al., 2003; He et al., 2019). Graduate students will complete an expanded battery comprising the Chinese Brief Interpersonal Competence Questionnaire (ICQ-15; Huang et al., 2023), Chinese 20-item Toronto Alexithymia Scale (TAS-20-C; Zhu et al., 2007), Chinese General Self-Efficacy Scale (GSES; Zhang and Schwarzer, 1995), Academic Self-Efficacy Scale (ASES; Liang, 2000), Chinese revised Achievement Goal Orientations Scale (AGOS; Liu and Guo, 2003), academic-achievement subscale of the Chinese Multidimensional-Multiattributional Causality Scale (MMCS; Lefcourt et al., 1979; Wang et al., 1999), and the Simplified Chinese Revised NEO Personality Inventory, for which the required authorization will be obtained before administration (NEO PI-R; Costa and McCrae, 1992; Yang et al., 1999).

#### 2.4.3 Physiological measurements

A multimodal approach will be used to gather objective physiological and neuro-functional data. Resting-state EEG will measure baseline neural activity. MRI and fNIRS will be employed to identify brain activation patterns and evaluate cortical blood oxygenation during verbal fluency and executive function tasks, respectively. Acoustic data will be recorded while participants read a standardized passage, and facial behavior will be recorded during open-ended questions for subsequent exploratory analysis. Previous work has shown that automatically coded facial actions and head movements vary with depression severity, although their screening value in university populations remains to be established (Girard et al., 2014). Additional assessments include eye-movement tracking to monitor visual processing and event-related potentials (ERPs), focusing on the P300 component, to evaluate cognitive processing. Lastly, heart rate variability (HRV) will be measured to assess autonomic regulation. Component-specific reasons for deferral or non-completion, together with the corresponding safety rationale and reasonable accommodations, are summarized in Supplementary Table 6.

#### 2.4.4 Planned biological sample collection

The biospecimen and multi-omics component is exploratory and has not yet been activated under the longitudinal program. Blood, urine, stool, and saliva are currently identified as candidate biological matrices. Before collection of the first sample of a given matrix at any participating site, a matrix-specific standard operating procedure will specify participant preparation, collection materials, sample volume, collection timing, processing intervals, centrifugation and aliquoting where applicable, transport conditions, storage temperature, permitted freeze–thaw cycles, and specimen-level quality-control criteria. No collection will begin until the relevant procedure has been finalized and approved. Participants may decline any biospecimen component without affecting their participation in the questionnaire, digital, or physiological components of the cohort. Before any omics assay is undertaken, an assay-specific protocol will define the eligible samples, laboratory platform, randomization across batches, technical controls and replicates, assay-failure criteria, preprocessing and normalization procedures, and statistical analysis. Transcriptomic, proteomic, metabolomic, lipidomic, microbiome, and other molecular analyses will therefore be treated as exploratory and will be conducted only after the corresponding procedures have been documented in a dated and version-controlled protocol amendment and submitted for ethics review where required.

#### 2.4.5 Planned embedded psychological interventions

All eight embedded intervention trials remain in the planning stage and have not commenced participant recruitment, enrolment, randomization, or intervention delivery. The present manuscript provides the master program framework rather than final trial-specific protocols. Across the currently planned interventions, delivery is expected to comprise 8–16 sessions over 4–12 weeks; this is a program-level planning range rather than a common fixed schedule. The trial-specific intervention arm and core components, format and setting, provisional dose, planned provider, comparator-development status, adherence and fidelity procedures, and manual-version status are summarized in Supplementary Table 7. Before activation and the first participant randomization of any trial, the final comparator and rationale, eligibility criteria, delivery format, dose, provider requirements, fidelity procedures, dated intervention manual, statistical analysis plan, ethics approval, and public registration record will be finalized and prospectively documented.

Students identified through cohort screening as potentially eligible for an embedded intervention trial will undergo trial-specific eligibility assessment. Each of the eight interventions will be evaluated in a separate two-arm, multi-center, parallel-group randomized controlled trial using 1:1 individual randomization. Eligibility will be determined using the symptom domain, severity range, safety criteria, contraindications, and other trial-specific criteria specified in Supplementary Table 3. Cohort screening and AI-generated model outputs will not by themselves establish final trial eligibility, select the trial entered, or determine randomized allocation.

If more than one embedded trial is recruiting at a given time, participants who meet the eligibility criteria for more than one available trial will be informed of the eligible options. The selected trial will be determined before randomization on the basis of trial availability and the participant’s informed preference and will be documented. A participant may enroll in only one intervention trial at a time. After trial-specific informed consent and the baseline assessment have been completed, participants will be individually randomized within the selected trial to the intervention or its trial-specific comparator. The comparator and its rationale will be finalized and prospectively documented in the trial registration, intervention manual, statistical analysis plan, and full trial-specific protocol before trial activation and the first participant randomization.

Randomization sequences will be computer-generated by an independent statistician and implemented through a central allocation system independently of the AI platform. Allocation will remain concealed from recruitment personnel until assignment is released. Because participants and intervention providers cannot be blinded to behavioral interventions, primary self-report outcomes will be collected using standardized electronic procedures without intervention-provider involvement. Investigators conducting researcher-administered assessments and the primary statistical analyst will remain blinded to allocation where feasible. Outcomes will be assessed at baseline, within 7 days after the scheduled final intervention session, and at the 3-month follow-up. The primary analysis of each trial will follow the intention-to-treat principle. Adherence, treatment fidelity, intervention discontinuation, withdrawal from outcome follow-up, and missing outcome data will be handled according to Supplementary Table 3.

### 2.5 Study status and protocol version

The initial cohort recruitment and university-student mental-health assessment at Henan Medical University were approved by the Ethics Review Committee of Henan Medical University on 8 April 2025 (approval no. XYLL-20250484), and implementation commenced in September 2025. Before enrolment, the ethics-approved document entitled “Intelligent Assessment of University Students’ Mental Health and the Effects of Group Psychological Interventions” was approved on 8 April 2025 and governed the Henan-site procedures. Its scope was limited to the initial Henan Medical University cohort recruitment and mental-health assessment.

The CAMPUS master protocol reported in this article was subsequently developed as a version-controlled document to describe the broader multiregional research program, including the planned expansion to additional universities, planned embedded intervention trials, multimodal assessments, and analytical procedures. It was not the pre-enrolment document governing the initial Henan-site implementation. Version 1.0 of the manuscript-associated master protocol was dated 10 May 2026. The current Version 2.0, dated 5 September 2026, incorporates revisions made during peer review. Before Version 1.0 was dated 10 May 2026, personnel developing the CAMPUS master protocol and the statistical and AI analysis plans had not received, viewed, exported, merged, summarized, or analyzed any individual-level or aggregate post-enrolment longitudinal-cohort outcome data. No data extracts, descriptive summaries, statistical outputs, or other reports containing post-enrolment cohort outcome information had been provided to those personnel. A site safety pathway for positive suicide-risk screens had been established before implementation and was separate from protocol-development and analytical activities. However, no participant generated a positive safety screen requiring case-level review or escalation before 10 May 2026. Thus, Version 1.0 was developed after cohort initiation but before inspection of post-enrolment cohort outcome data. Protocol versions and amendments are summarized in Supplementary Table 8. The first documentary record of key design and analytical decisions is provided in Supplementary Table 9.

### 2.6 Data analysis

#### 2.6.1 General statistical analysis

Data quality checks will include range and consistency checks, identification of duplicate records, and review of missing or implausible values. Extreme observations will not be removed solely because of their magnitude; suspected recording or measurement errors will be checked against the source data where possible. Variables will be transformed or standardized only when required for a particular analysis. Continuous variables will be summarized using means and SDs or medians and interquartile ranges, as appropriate, and categorical variables using counts and percentages. For design-based descriptive estimates, each participant’s base sampling weight will be calculated as the inverse of the realized probability of selection across the stratum, primary-sampling-unit, and individual-selection stages. Non-response adjustments will be estimated within pre-specified sampling cells defined by university, academic year, and major discipline, with additional administrative variables used where reliable population margins are available. Final weights will be calibrated to official university enrolment totals for the relevant strata where feasible. Variance estimation will account for strata, primary sampling units, and sampling weights. Weighted estimates will target the eligible enrolled student populations of the participating universities; they will not be interpreted as nationally representative estimates. Group comparisons will be treated as exploratory and conducted using regression models appropriate to the distribution of the outcome. Analyses will be performed using SPSS and R, with the exact software and package versions reported in the statistical analysis outputs.

Longitudinal analyses. Mixed-effects models will be used to estimate changes in mental health outcomes over time. The data-generating structure comprises repeated semester assessments within students, students within classes or administrative groups selected as primary sampling units, and primary sampling units within universities. For Objective A1, semester wave will be entered as a categorical fixed effect to allow a non-linear mean trajectory, with a pre-specified contrast based on the numerical semester index used to estimate the overall linear trend. Academic stage, sex, and university will also be included as fixed effects. The random-effects structure will comprise a participant-level random intercept and a random slope based on the numerical semester index, together with a random intercept for primary sampling unit. University will be treated as a fixed effect because the study includes only seven participating universities. A model treating university as a random effect will be examined as a sensitivity analysis.

For Objective A1, the primary analysis will use a weighted pseudo-likelihood mixed-effects model to estimate the population-average trajectory of repeated PHQ-9 scores across baseline and the nine scheduled semester follow-up waves. At baseline, each participant’s weight will equal the inverse of the realized probability of selection across the university, stratum, primary-sampling-unit, and individual-selection stages, with adjustment for baseline non-response within pre-specified sampling cells defined by university, academic year, and major discipline. Where feasible, the resulting weights will be calibrated to official university enrolment totals for the relevant strata.

For each follow-up wave, the baseline weight will be further adjusted for the estimated probability that PHQ-9 is observed at that wave, using pre-specified baseline characteristics and previously observed assessment information available before that wave. These observation-level weights will be used in the weighted mixed-effects model. Semester wave, university, sex, and academic stage will be included as fixed effects. The model will include participant-level random intercepts and random slopes for the numerical semester index, together with a random intercept for the primary sampling unit. University will be treated as a fixed effect. The sampling strata will be represented by the university-by-academic-year-by-major-discipline design strata. Ninety-five percent confidence intervals will be calculated using a design-based sandwich variance estimator that accounts for stratification and clustering within primary sampling units.

Pre-specified secondary models will examine effect modification by academic stage and sex through time × academic stage and time × sex interaction terms. Unweighted mixed-effects models will be reported as sensitivity analyses. The primary A1 analysis will use the untruncated final observation-level analysis weights. In a pre-specified sensitivity analysis, the final observation-level weights will be winsorized within each assessment wave, including baseline, at the 1st and 99th percentiles of the non-zero weight distribution. The truncated weights will then be rescaled within each wave to retain the same total weight as the corresponding original final weights. This sensitivity analysis will be conducted without reference to outcome values. These weighted estimates target the eligible enrolled student populations of the participating universities and will not be interpreted as nationally representative estimates.

For each embedded trial, the primary analysis will follow the intention-to-treat principle. Change from baseline in the trial-specific primary outcome at the post-intervention and 3-month assessments will be analyzed using a mixed-effects model including fixed effects for treatment group, assessment time, treatment × time, baseline primary-outcome score, and site, together with a participant-level random intercept. The primary treatment contrast will be the adjusted between-group difference in change from baseline at the pre-specified post-intervention assessment. Treatment effects at the 3-month follow-up and treatment × baseline-severity interactions will be considered exploratory. For interventions delivered in groups or by shared providers, the model will additionally account for the partially nested intervention-group or provider structure.

Intervention discontinuation will be distinguished from withdrawal from outcome follow-up. Participants who discontinue the assigned intervention will remain in the intention-to-treat population, will be analyzed according to their randomized group, and will be encouraged to complete all scheduled outcome assessments.

The primary model specifications will be defined before outcome analysis and will not be selected according to observed model fit. Model convergence, residual distributions, influential observations, and the adequacy of the random-effects structure will be examined graphically. Alternative covariance or random-effects specifications will be evaluated in sensitivity analyses if the primary model shows inadequate fit or fails to converge.

Missing data and attrition. The primary mixed-effects analyses will use maximum-likelihood estimation and all available outcome observations under a missing-at-random assumption. The amount and reasons for missing data will be reported by semester wave and, for the intervention trials, by randomized group. Wave-specific retention rates will be reported, and baseline characteristics of participants with and without subsequent outcome data will be compared using standardized differences.

Sensitivity analyses will include multilevel multiple imputation using models that reflect the hierarchical data structure and include the variables in the primary analysis, repeated outcomes, relevant interaction terms, and auxiliary variables associated with missingness. For the intervention trials, treatment allocation will be included in the imputation model. Departures from the missing-at-random assumption will be examined using delta-adjusted pattern-mixture models.

Variables unavailable by design. Measurements that were not scheduled for a particular participant group or assessment wave will not be imputed. Measurements unavailable by design will not be counted as missing follow-up observations or included in attrition denominators. The ICQ, TAS-20, GSES, AGOS, ASES, MMCS, and NEO PI-R are administered only to postgraduate students; analyses involving these instruments will therefore be restricted to the postgraduate subsample and treated as exploratory. Joint analyses of undergraduate and postgraduate students will use only instruments administered to both groups. The CTQ, which is collected at baseline only, will be analyzed as a time-invariant baseline variable.

Physiological, neuro-functional, and omics measures are collected only from participants selected for the multimodal subsample. Analyses involving these measures will be restricted to participants for whom the relevant modality was scheduled. Incidental missing values within a collected modality may be imputed according to the pre-specified analysis pipeline, but modalities that were not collected by design will not be imputed. In the prediction analyses described in Section 2.6.2, imputation, scaling, and other data-dependent preprocessing will be performed using the training data only.

Joint analyses and multimodal prediction models will be developed within pre-specified modality-availability blocks rather than assuming that all modalities are available for all participants. The core cohort-data block will include variables scheduled for the full cohort; postgraduate-only instruments will be analyzed within a separate postgraduate block; physiological and neuro-functional analyses will be restricted to the multimodal subsample; and MRI analyses will be restricted to participants for whom MRI was scheduled. Multi-omics data will be analyzed separately under Objective C3 and will not be included in the T1–T6 multimodal fusion framework. The eligible population, predictor set, sample size, and results will be reported separately for each relevant block.

#### 2.6.2 AI-assisted mental health screening and outcome-prediction analyses

The AI analyses will address questionnaire-defined screening and prediction tasks rather than clinical diagnosis. No structured diagnostic interview or independent clinical adjudication is planned. Tasks T1–T4 will classify concurrent PHQ-9 ≥ 10, GAD-7 ≥ 10, ISI ≥ 15, and any BSI-CV item scored above 0, respectively. T5 will predict the first subsequent PHQ-9 score ≥10 among participants with a baseline score <10; participants lost to follow-up before the relevant horizon without an observed event will be right-censored. T6 will predict continuous change in the trial-specific primary outcome measured within 7 days after the scheduled final intervention session. Participants with scores close to a threshold will be retained and classified according to the pre-specified rule. Full task definitions are provided in Supplementary Table 1. Any positive BSI-CV response will activate the safety assessment, irrespective of model output.

Candidate models will use eligible questionnaire, behavioral, physiological, and neuro-functional data according to task and modality availability. Inputs, preprocessing procedures, modality-specific encoders, and latent representations are specified in Supplementary Table 2. Each encoder will produce a fixed-dimensional latent representation that is projected to a common dimension and treated as a modality token. Available tokens within a pre-specified modality-availability block will be combined using multi-head self-attention across modalities, followed by a fusion multilayer perceptron and a task-specific prediction head. Sigmoid heads will be used for T1–T4, a discrete-time survival head for T5, and a linear head for the primary continuous T6 outcome. Multimodal models will be compared with unimodal models, regularized regression, and gradient-boosting models. Modalities not collected by design will not be imputed or synthetically reconstructed, and multi-omics data will be analyzed separately under Objective C3. The HRV and MRI feature-extraction procedures will be specified in dated, version-controlled computational protocols before any outcome-informed analysis is undertaken.

For concurrent classification tasks, the questionnaire defining the reference outcome, its constituent items, and variables directly derived from it will be excluded from the predictors. Longitudinal models will use only information collected before the predicted outcome. All data-dependent preprocessing, feature selection, class-imbalance handling, model fitting, hyper-parameter selection, and operating-threshold selection will be performed within the corresponding training partition. Data will be partitioned at the participant level so that all observations from the same participant remain within the same development or evaluation set. In longitudinal forecasting, earlier observations may predict later outcomes, but evaluation participants will remain separate from all model-development participants. Participant-grouped nested cross-validation will use inner folds for model selection and outer folds only for performance estimation. Temporal validation will evaluate later-wave data from participants held out from model development. Leave-one-site-out validation will train on six centers and evaluate the remaining center in turn. These procedures constitute internal validation and will not be described as independent external validation.

Performance measures will be selected according to outcome type, as specified in Supplementary Table 1. Predicted-probability thresholds will be selected using development data and locked before held-out evaluation. For T1–T3 and horizon-specific T5 classification, specificity will be maximized subject to a minimum sensitivity of 90%; the corresponding minimum for T4 will be 95%. Principal estimates will be reported with participant-level bootstrap 95% CIs. Center-specific estimates and exploratory subgroup analyses by sex and academic stage will be reported where sample size and outcome frequency permit stable estimation.

All models will be developed and evaluated offline. Their outputs will not determine clinical diagnosis, suicide-risk disposition, trial eligibility, randomized allocation, treatment selection, intervention intensity, or protocol modification. SHAP analyses of held-out predictions will be used to describe feature contributions but will not be interpreted as evidence of biomarker validity, biological mechanism, or causality. Reporting will follow the TRIPOD+AI recommendations (Collins et al., 2024).

#### 2.6.3 Trial-specific outcome-prediction analyses

The analysis framework will also be used to develop and evaluate exploratory models of trial-specific treatment outcomes using longitudinal self-report, behavioral, and physiological data collected before the scheduled primary outcome assessment. The primary prediction target will be continuous change from baseline in the trial-specific primary outcome measured within 7 days after the scheduled final intervention session, with 3-month outcomes examined secondarily. Recurrent neural networks, including long short-term memory networks, and time-series Transformer models will be compared with pre-specified benchmark models. Model development and evaluation will be conducted offline. Model predictions and generative summaries will not be used to determine trial eligibility, randomized allocation, treatment selection, intervention intensity, intervention delivery, or modification of intervention protocols during the embedded trials. Any future use of these models for clinical decision support would require prospective external validation and separate evaluation of clinical utility, safety, ethics, and implementation (Figure 2).

**Figure 2:**
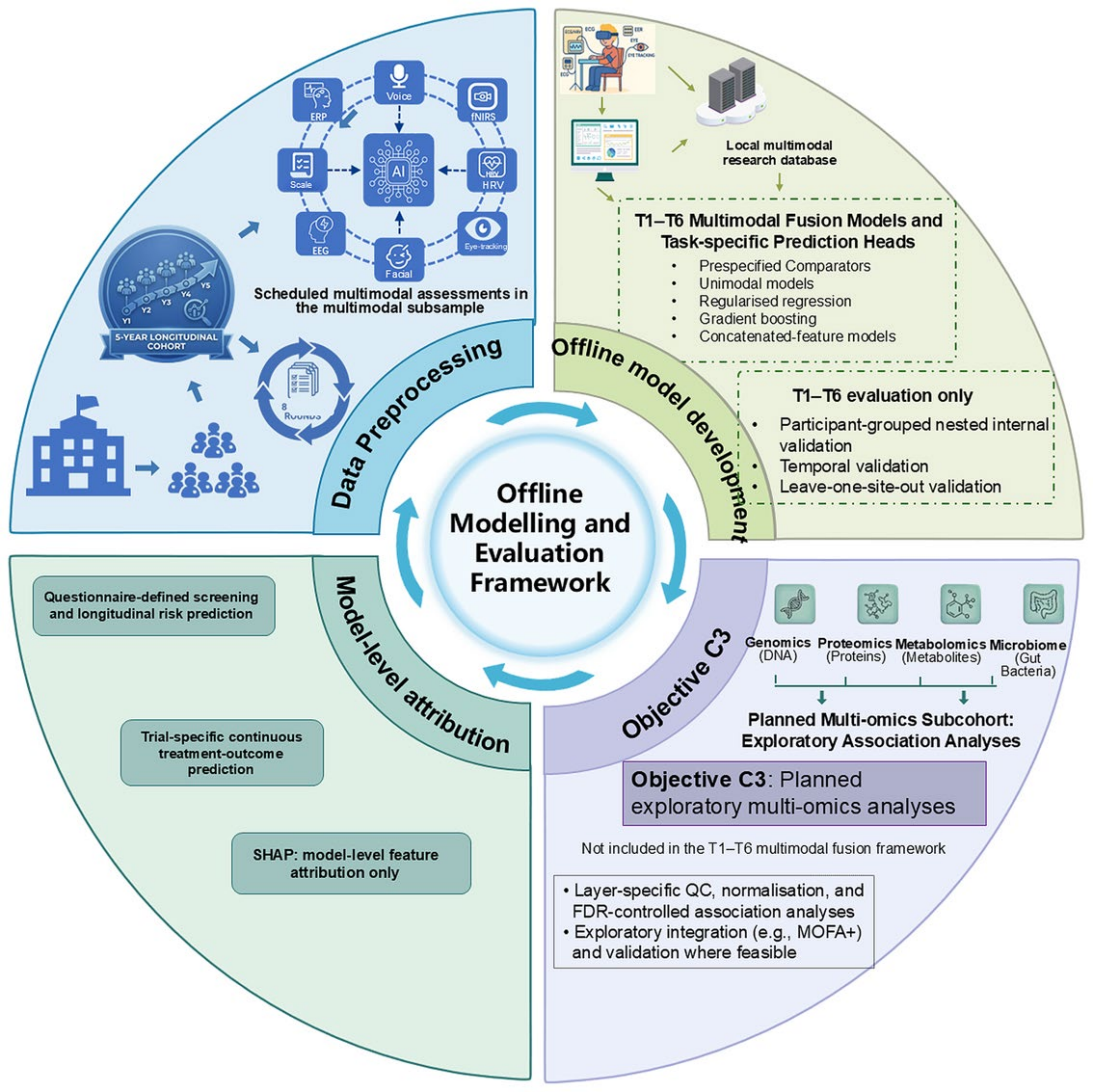
Overview of the CAMPUS analytical framework. The T1–T6 branch represents the planned offline screening and prediction analyses using questionnaire, behavioral, physiological, and neurofunctional data. Objective C3 represents separate exploratory multi-omics analyses and is not included in the T1–T6 multimodal fusion framework. FDR, false-discovery rate; MOFA+, multi-omics factor analysis+.

For interventions delivered in groups or by shared providers, the primary analysis will reflect the partially nested design. Intervention-group or provider effects will be represented by an arm-specific random effect where supported by the realized design, while participants in an ungrouped comparator arm will be treated as singleton clusters. Site will be included as a fixed effect, and participant-level random effects will account for repeated measurements. The intervention-group and provider intra-class correlation coefficients will be reported with CIs. If the number of realized groups or providers is insufficient for stable random-effect estimation, pre-specified small-sample or cluster-robust sensitivity analyses will be used.

#### 2.6.4 Exploratory bioinformatic and multi-omics analyses

The multi-omics analyses are designed to examine molecular correlates of mental-health outcomes and are organized around five pre-specified questions: (Q1) Which molecular features are associated with depressive symptom severity at baseline and over follow-up? (Q2) Which baseline molecular features are associated with the first subsequent PHQ-9 score ≥10 among participants with PHQ-9 < 10 at baseline? (Q3) Are multi-omics profiles associated with estimated depressive-symptom trajectories? (Q4) Which biological pathways are enriched among symptom-associated molecular features? (Q5) Among trial participants with the required baseline biospecimens, which baseline molecular features are associated with continuous change from baseline in the trial-specific primary outcome at the scheduled post-intervention assessment? The eligible population, available omics layers, outcome definition, assessment timing, covariates, and analysis method for each question are specified in Supplementary Table 5. Previous case–control work has suggested that integrating transcriptomic and epigenomic layers may improve MDD classification compared with individual molecular layers, although independent validation is required (Mokhtari et al., 2025). This evidence informs the exploratory integration strategy but does not provide the basis for the CAMPUS sample-size calculation. All analyses in this component are exploratory and hypothesis-generating and will not be interpreted as confirmatory, mechanistic, or causal.

Raw data from each omics layer will undergo assay-specific sample and feature quality control, filtering, normalization, and assessment of technical variation. Batch, plate, sequencing run, study center, and other relevant technical variables will be recorded and addressed using methods appropriate to the assay. Where biological and technical variables are partly confounded, sensitivity analyses will be used to assess the effect of alternative adjustment strategies. The number of samples and features retained at each processing stage, together with the software, parameter settings, and reference database versions, will be reported.

Missing-value handling will be specified separately for each omics layer according to the measurement process and the likely source of missingness. Values that are missing because an omics layer was not collected by design will not be imputed. For prediction analyses, imputation, normalization, batch adjustment, and feature selection will be performed within the training partitions to prevent information leakage. Sensitivity analyses will compare reasonable layer-specific preprocessing choices where these could materially affect the findings.

For Q1, feature-level associations with continuous symptom scores will be estimated using regression or mixed-effects models appropriate to the outcome and measurement schedule. Models will adjust for a pre-specified set of demographic, clinical, technical, and assay-specific covariates, including age, sex, study center, and batch, with additional variables such as body mass index, smoking, medication use, or estimated cell composition included where relevant to the omics layer. For Q2, prediction models will be developed and evaluated according to the procedures described in Section 2.6.2, with analyses restricted to participants with PHQ-9 < 10 at baseline. The outcome will be the first subsequent PHQ-9 score ≥10 during the pre-specified follow-up horizon, with loss to follow-up handled as right censoring. For Q3, MOFA+ will be used as a latent-factor method to derive factors representing variation shared across or specific to individual omics layers. Associations between these factors and symptom trajectories will be evaluated after the trajectories have been estimated from the longitudinal clinical data without using omics information. Supervised sparse partial least-squares methods will be used only for separately pre-specified regression or classification tasks and will be evaluated using participant-level resampling and held-out data. For Q4, pathway and gene-set enrichment analyses will be performed using KEGG, Reactome, and Gene Ontology. Each analysis will specify the feature-selection rule, identifier mapping procedure, background feature universe, database release, statistical test, and multiple-testing correction. For Q5, associations between baseline molecular features and continuous change from baseline in the trial-specific primary outcome will be analyzed separately within each embedded trial. Models will include treatment group, the baseline value of the trial-specific primary outcome, study site, and relevant demographic and technical covariates. Feature × treatment interactions may be examined as exploratory analyses of treatment-effect heterogeneity where the available sample size permits.

False-discovery-rate (FDR) correction will be applied within each pre-specified family of feature-level tests, defined by research question and omics layer and, for Q5, by embedded trial. The adjusted significance threshold and the number of tests in each family will be reported. Where sample size permits, selected findings will undergo internal validation in a held-out participant subset. Evidence from a different omics layer will be described as cross-omics convergence rather than replication, and findings without validation in an independent sample will be reported as discovery findings requiring further replication.

SHAP may be used in the prediction analyses to summarize the contribution of molecular features to model predictions. SHAP values will be interpreted only as model-level feature attributions and will not be considered evidence of clinical importance, biomarker validity, biological mechanism, or causality. Mendelian randomization is outside the scope of the present protocol and will not be used to support causal claims in the pre-specified CAMPUS analyses. The HRV and MRI computational specifications will be dated, version-controlled, and locked before the corresponding features are linked to study outcomes or used in model-performance evaluation; post-lock modifications will be documented and treated as exploratory.

### 2.7 Study status and operational monitoring

Longitudinal cohort enrolment commenced at Henan Medical University in September 2025. Activation of each additional university will require local ethics approval and completion of site-specific preparations. This article describes the study protocol and does not report participant-level or aggregate findings from the earlier developmental activities or the longitudinal cohort.

During longitudinal follow-up, operational indicators will be monitored by center and assessment wave. Recruitment-flow indicators will also be reported by academic year and major discipline where cell sizes permit, with the denominator for each rate stated explicitly. Indicators will include the numbers approached, screened, eligible and enrolled; recruitment and assessment-completion rates; modality-specific attempted, completed and quality-control-eligible assessments; technical-failure rates; assessment duration; participant-reported burden or acceptability where collected; reasons for non-participation and withdrawal; wave-specific retention; intervention attendance and discontinuation; and adverse events in the embedded trials. These indicators will be reported descriptively in subsequent publications; no formal go/no-go feasibility thresholds are pre-specified. The study timeline and planned implementation are summarized in Figure 3.

**Figure 3:**
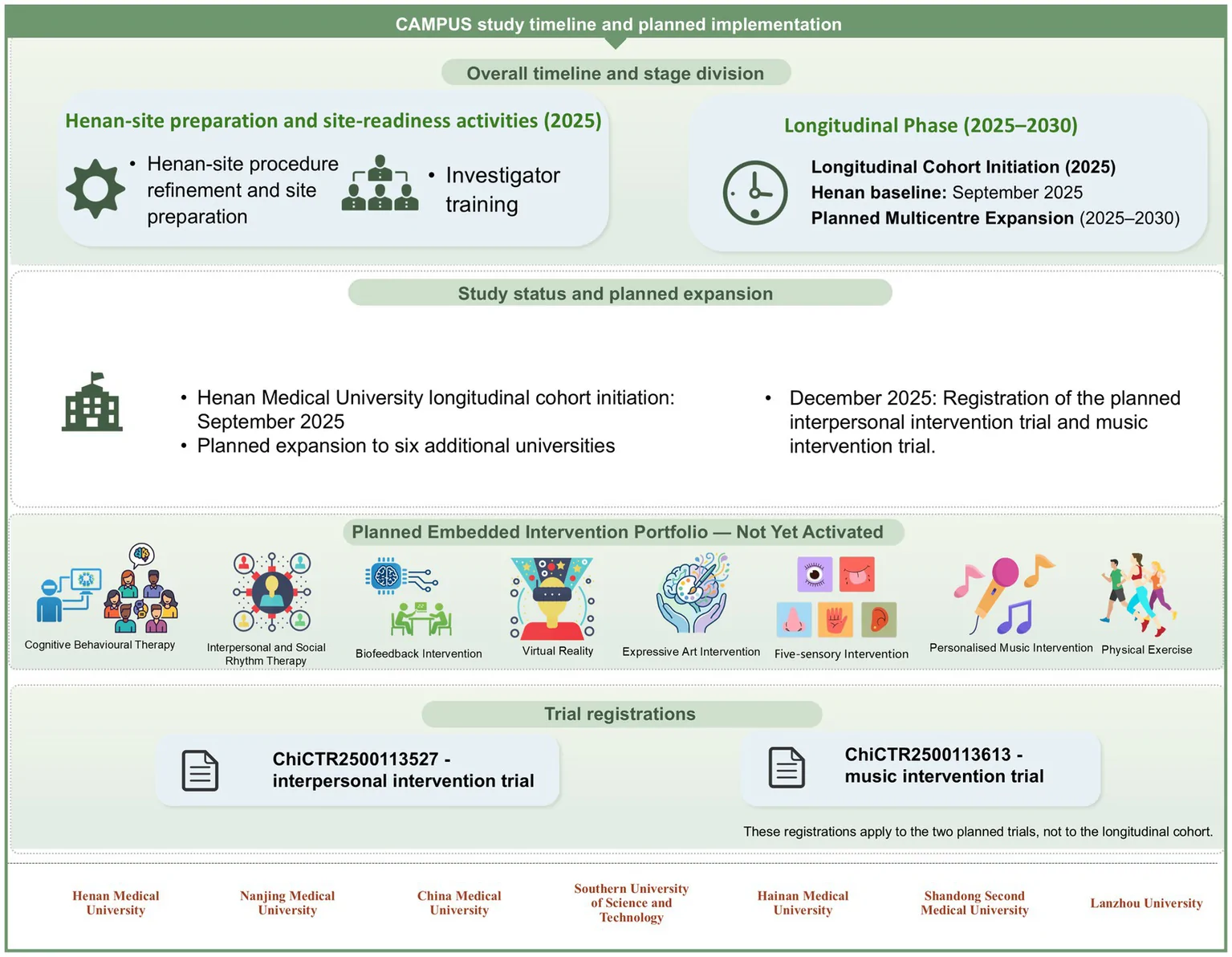
CAMPUS study timeline and planned implementation. ChiCTR, Chinese Clinical Trial Registry.

## 3 Discussion

CAMPUS is a large-scale, multi-center, multimodal, longitudinal research program conducted across seven Chinese universities. It is designed to integrate longitudinal cohort follow-up, embedded randomized trials, multimodal assessments, and exploratory AI and multi-omics analyses within a coordinated research program. It is designed to integrate multidimensional assessment with AI-assisted mental-health screening and planned intervention formats targeting depressive symptoms, anxiety symptoms, insomnia, and stress-related concerns. By linking psychological, physiological, and biological data, CAMPUS provides a framework for the exploratory evaluation of multidimensional screening and treatment-outcome prediction. The planned biospecimen component may support exploratory multi-omics analyses after the relevant matrix- and assay-specific procedures have been finalized and approved. These analyses will examine molecular correlates of longitudinal mental-health outcomes but will not be interpreted as establishing biomarkers, mechanisms, or causal relationships. Whether these data improve model generalizability or clinical usefulness remains to be demonstrated. Together, these components provide a research framework for prospectively evaluating multidimensional screening, trial-specific interventions, and exploratory molecular correlates. Whether this framework can be implemented effectively or scaled to other university settings will require separate prospective evaluation.

A key strength of the CAMPUS study is its design, which combines comprehensive multidimensional assessment with planned longitudinal follow-up, embedded trials, and exploratory multimodal analyses. Single-modality approaches may not capture information available across complementary behavioral, physiological, and neuro-functional data sources. Whether integrating these data sources improves screening performance will be evaluated prospectively against pre-specified unimodal and concatenated-feature benchmarks. Multimodal AI approaches may capture complementary information across data types, but their incremental performance, calibration, and transportability vary across tasks and settings and require direct comparison with appropriate unimodal benchmarks (Schouten et al., 2025). School-based programs such as Canada’s EMPATHY program have reported reductions in suicidality, depression, and anxiety following implementation (Silverstone et al., 2017), although the program mainly relied on self-reported psychometric measures and did not include linked neuroimaging or multi-omics assessments. Similarly, StudentLife demonstrated the feasibility of using smartphone-based passive sensing to examine behavioral, mental-health, and academic patterns among university students (Wang et al., 2014), but it did not include linked neuroimaging, biospecimens, or multi-omics assays. CAMPUS will prospectively evaluate whether these additional modalities improve performance over pre-specified unimodal and concatenated-feature benchmarks. CAMPUS is designed to examine whether a comprehensive multidimensional screening framework can address some of these limitations. Pre-specified Transformer-based multimodal fusion models will be used to examine interactions across the available data types. This combination of data sources will enable prospective evaluation of whether multimodal models improve screening performance over pre-specified unimodal and concatenated-feature benchmarks within the participating universities. This multidimensional platform enables prospective evaluation of whether the additional modalities improve screening performance within the participating universities. Its transportability, clinical utility, and value for informing intervention pathways will require further evaluation.

Beyond multidimensional screening, CAMPUS brings longitudinal assessment and eight planned embedded intervention trials together within a coordinated research platform. Evidence supports several intervention formats relevant to young people and university students, including indicated prevention programs for at-risk higher-education students (Conley et al., 2017), internet-based self-help for depression in adolescents and young adults (Ma et al., 2024), internet-delivered CBT for anxiety among university students (Oliveira et al., 2023), and AI-based conversational agents for reducing depressive symptoms and psychological distress (Li et al., 2023). Each CAMPUS intervention will be evaluated separately in an embedded randomized trial with its own eligibility criteria, comparator, primary outcome, and analysis population. AI models of trial-specific treatment outcomes will be developed and evaluated offline. Model predictions will not determine trial eligibility, randomized allocation, treatment selection, intervention intensity, or modification of intervention protocols during the embedded trials.

The CAMPUS program includes a range of individual and group-based intervention formats, including IPSRT, CBT, music, physical exercise, virtual reality, expressive art, biofeedback, and five-sensory stress-regulation interventions. Each intervention will be evaluated in a separate randomized trial with its own eligibility criteria, comparator, primary outcome, and analysis population. Students identified through cohort screening as potentially eligible will undergo trial-specific eligibility assessment, and entry into a recruiting trial will be determined before randomization on the basis of trial availability and the participant’s informed preference. AI models and physiological profiles will not determine trial entry, treatment selection, or intervention allocation. The longitudinal psychological, physiological, and biological data will instead be used for exploratory offline analyses of screening performance, treatment-outcome prediction, and molecular correlates.

Previous work has reported associations among anhedonia, inflammatory markers, and functional connectivity in young people with recent-onset mental disorders (Lalousis et al., 2024). These findings provide a rationale for exploratory molecular analyses but do not establish validated biomarkers or biological mechanisms. The CAMPUS multi-omics analyses will examine available genomic, DNA-methylation, metabolomic, and microbiome data in relation to longitudinal clinical phenotypes. Transcriptomic, proteomic, lipidomic, and other additional omics layers will be included only if they are generated under pre-specified layer-specific protocols. These associations will be treated as discovery findings unless reproduced in an independent sample. Associations that are independently replicated and demonstrate adequate analytical validity and incremental predictive value may subsequently be evaluated as candidate biomarkers. Functional or experimental studies would be required to establish biological mechanisms, and causal conclusions would require a separately specified causal-inference design and its associated assumptions. SHAP values will be used only to describe how features contribute to predictions within a fitted model; they will not be considered evidence of biomarker validity, biological mechanism, or causality. CAMPUS may therefore generate molecular and model-based hypotheses for further investigation but will not use feature attribution or observational molecular associations to support mechanistic or causal claims.

### 3.1 Limitations

Several limitations should be acknowledged. First, the participating universities were selected through research collaborations rather than probability sampling from a national sampling frame. Despite their multiregional distribution, the cohort is not nationally representative, and the predominantly Han Chinese sample may further limit generalizability to other institutions and student populations. Second, the five-year follow-up is subject to attrition; retention is monitored by wave and missing-data strategies are pre-specified. Third, participation in high-burden modalities (MRI, biospecimen collection) may differ systematically by participant characteristics, and the biological subsamples may therefore be subject to selection bias; this will be examined by comparing subsample participants with the full cohort on baseline variables. Fourth, physiological measures are acquired with center-specific devices, and although acquisition and quality-control procedures are harmonized across centers, residual center and device heterogeneity may affect pooled estimates; site effects are addressed in the pre-specified models. Fifth, neuro-functional assessments are conducted in controlled laboratory environments, which may not fully capture real-world stressors. Sixth, multimodal missingness arises because modality blocks are collected in specific subsamples; structurally missing data are never imputed, and incidental missingness is handled with the pre-specified strategies. Seventh, the exclusion of students with diagnosed psychiatric disorders limits the examination of the full continuum of symptom severity. Eighth, temporal changes in protocols, questionnaire versions, or platform components during the five-year program are possible and will be documented as protocol amendments in the trial registry. Ninth, algorithmic bias is a recognized risk, including differential model performance across subgroups defined by sex, academic stage, or center. Performance will therefore be reported in pre-specified subgroups where sample sizes and outcome-event counts permit stable estimation. Temporal validation and leave-one-site-out validation will be used to examine model transportability within the participating universities; however, these procedures constitute internal validation and do not establish transportability, clinical utility, or implementation feasibility in independent external settings.

### 3.2 Protocol version and amendments

This article reports CAMPUS master protocol Version 2.0, dated 5 September 2026. It supersedes Version 1.0 of the manuscript-associated CAMPUS master protocol and incorporates clarifications made during peer review. It is distinct from the pre-enrolment ethics-approved cohort documentation that governed the initial Henan Medical University cohort enrolment. Any substantive amendment after publication will be dated and versioned in the protocol amendment record and approved by the relevant ethics committee where required. Where an amendment concerns a registered embedded trial, the corresponding registry record will also be updated before implementation. The chronology of the ethics-approved cohort documentation and manuscript-associated protocol versions is provided in Supplementary Table 8. Supplementary Table 9 identifies the first documentary record of key design and analytical decisions.

### 3.3 Ethics and dissemination

The seven pilot survey waves conducted between March 2020 and September 2024 and the associated pilot intervention programs were approved by the Ethics Review Committee of Henan Medical University (approval no. XYLL-20200484). Oral informed consent was obtained for participation in the pilot surveys, whereas written informed consent was obtained before participation in a pilot intervention program. The initial Henan Medical University cohort recruitment and university-student mental-health assessment were approved by the Ethics Review Committee of Henan Medical University on 8 April 2025 (approval no. XYLL-20250484), before implementation commenced in September 2025. The approval was based on the pre-enrolment Henan-site document identified in Supplementary Table 8. All longitudinal-cohort participants are aged 18–30 years, inclusive, and provide written informed consent before enrolment. Separate written trial-specific informed consent will be obtained before enrolment and randomization in an embedded intervention trial. Each additional participating university will obtain approval from its responsible ethics committee before site activation, participant enrolment, or data collection.

Written consent for the longitudinal cohort will cover the core questionnaire assessments and longitudinal follow-up. Voice recording, facial-expression video recording, physiological and neuro-functional assessments, MRI, and future biospecimen collection for genetic and other multi-omics analyses will be subject to separate component-specific consent or consent options, as approved by the responsible ethics committee. Participants may decline or discontinue any optional component without affecting their continued participation in the core cohort. The biospecimen component has not yet been activated and will not commence until the matrix-specific procedures, consent materials, and required ethics amendments have been approved. Separate written trial-specific consent will be obtained before enrolment and randomization in any embedded intervention trial.

Research data and biospecimens will be assigned coded study identifiers. Direct identifiers and the linkage key will be stored separately from the research dataset in access-restricted, institutionally managed systems. Access to identifiable information, raw biometric data, genomic data, biospecimens, and the linkage key will be limited to authorized personnel according to their study roles and will be subject to institutional oversight. Data transfers between participating sites and the coordinating center will use approved secure transmission procedures. Requests for access to biospecimens or sensitive individual-level data will require scientific and institutional review and must be consistent with the approved consent provisions, ethics approvals, and applicable data-use agreements. Retention and destruction will follow the approved protocol, consent materials, institutional policy, and applicable legal requirements.

Raw voice recordings, facial-expression videos, imaging data, genomic data, and other data carrying a material risk of re-identification will not be deposited in public repositories. Derived features, trained model weights, embeddings, and other model artifacts will undergo disclosure-risk review before sharing because they may retain information about the training data. Publicly released code and documentation will not contain participant-level sensitive data or linkage information. AI models will be developed and evaluated offline and will not determine clinical diagnosis, suicide-risk disposition, trial eligibility, randomized allocation, treatment selection, intervention intensity, or intervention delivery.

## 4 Conclusion

CAMPUS is a multiregional research program comprising a five-year longitudinal cohort, eight planned embedded randomized trials, and exploratory multimodal AI and multi-omics analyses. It is designed to examine longitudinal mental-health outcomes, evaluate individual interventions, and develop and internally evaluate offline models for questionnaire-defined screening and treatment-outcome prediction. Intervention efficacy, model clinical utility and transportability, and the generalizability of the findings remain to be established through prospective evaluation.
