# Supplementary material for: Comprehensive artificial intelligence-assisted mental health program for university students in China (CAMPUS): study protocol for a multi-center prospective cohort with planned embedded randomized intervention trials
Source: Front Psychol. 2026 Oct 14;17:1877887. doi: 10.3389/fpsyg.2026.1877887 (PMC13713620; doi:10.3389/fpsyg.2026.1877887)
Supplement: Supplementary file 1 [file Supplementary_file_1.docx]

**Supplementary Material**

**Supplementary Methods**

**1. Psychological Assessments**

***Patient Health Questionnaire-9 (PHQ-9)***

The Patient Health Questionnaire-9 (PHQ-9) is a self-administered instrument comprising nine items, developed to assess depressive symptoms. Each item is evaluated on a four-point Likert scale ranging from 0 ("not at all") to 3 ("nearly every day"), yielding a cumulative score between 0 and 27, with higher scores indicating more severe depressive states. Symptom severity is conventionally classified as follows: 0–4 denotes no depression, 5–9 indicates mild depression, 10–14 represents moderate depression, 15–19 corresponds to moderately severe depression, and 20–27 indicates severe depression.

***Generalised Anxiety Disorder-7 (GAD-7)***

The Generalised Anxiety Disorder-7 (GAD-7) is a self-reported screening tool consisting of seven items, designed to screen for potential cases and evaluate the severity of anxiety symptoms. Items are rated from 0 ("not at all") to 3 ("nearly every day"), with total scores ranging from 0 to 21. A score of 0 to 4 indicates no anxiety, 5 to 9 indicates mild anxiety, 10 to 14 indicates moderate anxiety, and 15 to 21 indicates severe anxiety.

***Insomnia Severity Index (ISI)***

The Insomnia Severity Index (ISI) is a seven-item questionnaire designed to evaluate the severity and impact of insomnia symptoms. Each item is scored from 0 ("no problem") to 4 ("very severe problem"), resulting in a total score range of 0 to 28. Scores of 0 to 7 suggest no clinically significant insomnia, 8 to 14 suggest subthreshold insomnia, 15 to 21 indicate moderate insomnia, and 22 to 28 reflect severe insomnia.

***10-Item Perceived Stress Scale (PSS-10)***

The PSS-10 is a 10-item instrument that assesses the extent to which individuals perceive their lives as stressful. Items are rated on a five-point scale from 0 (“never”) to 4 (“very often”), yielding a total score ranging from 0 to 40, with higher scores indicating greater perceived stress. No validated diagnostic cut-off is prespecified in CAMPUS. Scores of 14–26 are used only as a study-defined eligibility range for the B5 biofeedback and B8 five-sensory stress-regulation trials and will not be interpreted as a diagnostic category (Cohen et al., 1983).

***Chinese Version of the Beck Scale for Suicide Ideation (BSI-CV)***

The Chinese version of the Beck Scale for Suicide Ideation (BSI-CV) is a 19-item self-report measure of suicidal ideation and suicidal tendency. The first five items assess suicidal ideation, and the remaining 14 items assess suicidal tendency. Each item is scored from 0 to 2, yielding a total score ranging from 0 to 38, with higher scores indicating more severe suicidal ideation and suicidal tendency. Its reliability and validity among Chinese university students have been established (Li et al., 2011).

***Snaith–Hamilton Pleasure Scale (SHAPS)***

The Chinese version of the Snaith-Hamilton Pleasure Scale (SHAPS) is a 14-item measure of current hedonic capacity (Snaith et al., 1995; Liu et al., 2012). Items are scored on a 4-point scale, yielding a total score of 0-14, with higher scores indicating greater anhedonia. SHAPS scores will be analysed as continuous measures. No diagnostic threshold is prespecified; a score of ≥3 may be reported only as a descriptive operational classification.

***Temporal Experience of Pleasure Scale (TEPS)***

The Chinese 20-item Temporal Experience of Pleasure Scale (TEPS) assesses anticipatory and consummatory pleasure (Chan et al., 2012). Items are rated on a 6-point Likert scale, yielding a total score of 20-120; higher scores indicate greater capacity for pleasure. TEPS scores will be analysed as continuous measures.

***Mobile Phone Addiction Tendency Scale (MPATS)***

The Mobile Phone Addiction Tendency Scale for College Students (MPATS) is a 16-item Chinese instrument assessing withdrawal symptoms, salience, social comfort, and mood changes (Xiong et al., 2012). Items are rated from 1 (“completely disagree”) to 5 (“completely agree”), producing a total score of 16-80. Higher scores indicate greater mobile-phone addiction tendency. No diagnostic cut-off is prespecified; the total score will be analysed continuously.

***Childhood Trauma Questionnaire (CTQ)***

The Chinese Childhood Trauma Questionnaire-Short Form (CTQ-SF) is a 28-item retrospective measure of childhood maltreatment (Bernstein et al., 2003; He et al., 2019). The five clinical subscales assess emotional abuse, physical abuse, sexual abuse, emotional neglect, and physical neglect. The total score is calculated from the 25 clinical items; the three minimisation/denial items will be considered separately. CTQ total and subscale scores will be analysed as continuous baseline variables. No CTQ threshold will determine trial eligibility, clinical diagnosis, or a primary outcome. Any exploratory descriptive categorisation will be prespecified and identified as an operational rule.

***Interpersonal Competence Questionnaire (ICQ)***

The Chinese Brief Interpersonal Competence Questionnaire (ICQ-15) is a 15-item self-report measure of initiating relationships, negative assertion, self-disclosure, emotional support, and conflict management (Huang et al., 2023). Items are rated on a 5-point scale, with higher scores indicating greater interpersonal competence. Scores will be analysed continuously.

***Toronto Alexithymia Scale (TAS-20)***

The Chinese 20-item Toronto Alexithymia Scale (TAS-20-C) assesses difficulty identifying feelings, difficulty describing feelings, and externally oriented thinking (Zhu et al., 2007). Items are rated from 1 (“strongly disagree”) to 5 (“strongly agree”). Total and subscale scores will be analysed continuously. For descriptive reporting only, TAS-20 total scores will be grouped as ≤51, 52–60, and ≥61. These are study-defined operational categories and will not be interpreted as diagnostic classifications, validated clinical thresholds, trial-eligibility criteria, or AI reference labels. Analyses involving TAS-20 will use the continuous total score where applicable.

***General Self-Efficacy Scale (GSES)***

The Chinese General Self-Efficacy Scale (GSES) is a 10-item measure of general perceived self-efficacy (Zhang and Schwarzer, 1995). Items are rated on a 4-point scale, and higher scores indicate greater perceived self-efficacy. Scores will be analysed continuously.

***Academic Self-Efficacy Scale (ASES)***

The Academic Self-Efficacy Scale (ASES) developed for Chinese university students comprises 22 items, including 11 learning-ability self-efficacy items and 11 learning-behaviour self-efficacy items (Liang, 2000). Items are rated on a 5-point scale. Total and subscale scores will be analysed continuously.

***Achievement Goals Orientation Scale (AGOS)***

The Chinese revised Achievement Goal Orientations Scale (AGOS) is a 29-item instrument assessing mastery-approach, performance-approach, mastery-avoidance, and performance-avoidance goals (Liu and Guo, 2003). Items are rated on a 5-point scale from 1 (“completely disagree”) to 5 (“completely agree”). Total and subscale scores will be analysed continuously.

***Multidimensional Multiattributional Causality Scale (MMCS)***

The academic-achievement subscale of the Chinese Multidimensional-Multiattributional Causality Scale (MMCS) will be used to assess causal attributions for academic outcomes (Lefcourt et al., 1979; Wang et al., 1999). This 24-item subscale assesses attributions to ability, effort, context, and luck. Items are rated from 0 (“strongly disagree”) to 4 (“strongly agree”). Internal-attribution scores are derived from ability and effort items, whereas external-attribution scores are derived from context and luck items. Scores will be analysed continuously.

***Revised NEO Personality Inventory (NEO PI-R)***

Personality traits will be assessed using the authorised Simplified Chinese Revised NEO Personality Inventory (NEO PI-R), a 240-item measure of Neuroticism, Extraversion, Openness, Agreeableness, and Conscientiousness and their 30 facets (Costa and McCrae, 1992; Yang et al., 1999). Items are rated on a 5-point scale from 0 to 4. Domain and facet scores will be analysed continuously.

**2. Physiological Measurements**

***Electroencephalography (EEG)***

Using Nexus-32 hardware and Biotrace software (MindMedia BV, Herten, the Netherlands), a 5-minute resting-state EEG recording will be obtained with participants’ eyes closed. EEG signals will be sampled at 512 Hz from 19 scalp electrodes positioned according to the international 10–20 system,Fp1, Fp2, F3, F4, F7, F8, C3, C4, T3, T4, T5, T6, P3, P4, O1, O2, Fz, Cz, and Pz, with A1 and A2 recorded as mastoid reference channels. Electrode impedances will be maintained below 5 kΩ before recording. The first 20 seconds will be discarded, and the subsequent fixed 150-second interval from 20 to 170 seconds will constitute the prespecified analysis window. No alternative segment will be selected on the basis of visual signal quality.

The fixed analysis window will be filtered using a 0.5–45 Hz finite impulse response bandpass filter. A scalp channel will be classified as bad if more than 20% of samples within the fixed analysis window have an absolute amplitude exceeding 100 µV. A recording will be excluded from the primary EEG analysis if four or more of the 19 scalp channels are classified as bad. Otherwise, bad channels will be temporarily removed, and the retained channels will be re-referenced to the linked mastoids. Extended Infomax independent component analysis will then be performed using EEGLAB. Components will be classified using ICLabel, and components assigned a probability of ≥0.90 to either the Eye or Muscle category will be removed. Previously removed bad channels will subsequently be reconstructed using spherical spline interpolation.

Power spectral density will be calculated using the fast Fourier transform to estimate absolute and relative power in the delta (1–4 Hz), theta (4–8 Hz), alpha (8–13 Hz), beta (13–30 Hz), and gamma (30–45 Hz) bands. Functional connectivity will be estimated using weighted phase-lag index matrices for each frequency band, from which prespecified global and local graph-theoretical measures will be derived using the Brain Connectivity Toolbox.

***Magnetic Resonance Imaging (MRI)***

MRI will be undertaken at all seven participating centres through local radiology departments or hospital partners using harmonised 3.0-T acquisition and quality-control procedures. The procedures described below apply to the Henan Medical University site; corresponding scanner-specific parameters and harmonisation procedures will be documented for the other participating centres before site activation. Magnetic resonance imaging (MRI) scans will be conducted in the Department of Radiology at the Second Affiliated Hospital of Henan Medical University. The scanning procedures will be performed by radiologists who are proficient in MRI operations. A 3.0 T MRI scanner will be used for the scans. Structural T1-weighted MRI images will be acquired using a 3D MPRAGE sequence with the following parameters: field of view (FOV) = 224 × 224 mm, flip angle = 8°, repetition time/echo time/inversion time (TR/TE/TI) = 2400/2.14/1000 ms, voxel size = 0.7 mm isotropic, bandwidth (BW) = 210 Hz/pixel, integrated parallel acquisition techniques (iPAT) = 2. T2-weighted images will be obtained using a 3D T2-SPACE sequence, with the following parameters: TR/TE = 3200/565 ms, flip angle variable, FOV = 224 × 224 mm, voxel size = 0.7 mm isotropic, BW = 755 Hz/pixel, iPAT = 2. Resting-state MRI data will be acquired using a gradient echo planar imaging sequence with the following parameters: TR = 2000 ms, TE = 30 ms, FOV = 220 × 220 mm, slice thickness = 4 mm, inter-slice gap = 0.6 mm, number of slices = 33, in-plane resolution = 64 × 64, flip angle = 90°.

The acquisition parameters reported above apply to the Henan Medical University site. Before activation of each additional MRI site, scanner-specific acquisition parameters, compatibility checks, and cross-site quality-control and harmonisation procedures will be documented. Before any outcome-informed MRI analysis, a dated and version-controlled MRI computational specification will define the software and version; structural and resting-state functional preprocessing workflows; template space and atlas/parcellation; image- and participant-level quality-control criteria; motion handling and exclusion thresholds; nuisance-regression and temporal-filtering procedures; regional structural features; connectivity definition; harmonisation method and its fitting strategy; and feature standardisation. No MRI feature selection, outcome association analysis, predictive-model fitting, threshold selection, or performance evaluation will be undertaken before this specification is locked. The specification will be locked before any MRI feature is linked to a questionnaire-defined outcome, trial outcome, or model-performance evaluation. No observed association with an outcome, model-performance result, or threshold-performance result will be used to select or modify the primary image-processing workflow, quality-control thresholds, atlas or parcellation, motion-handling procedure, harmonisation method, or primary feature set. Any post-lock modification will be documented in a dated version-controlled amendment and reported as exploratory; it will not replace the prespecified primary analysis.

***Functional Near-Infrared Spectroscopy (fNIRS)***

The functional near-infrared spectroscopy (fNIRS) experiment will be conducted in a controlled environment characterised by darkness and the absence of noise. Participants will be instructed to minimise head movements and maintain emotional stability throughout the experiment. A 48-channel fNIRS system (NirScan, Danyang Huichuang Medical Equipment Co., Ltd, Danyang, China) will be employed in this study. The Chinese version of the Verbal Fluency Test (VFT) will be employed to assess verbal fluency, working memory, verbal recall, attention, and retrieval. The VFT task consists of three distinct phases: a 30-second pre-task baseline period, a 60-second VFT task period, and a 70-second post-task period. During the 30-second pre-task baseline phase, participants will be instructed to repeatedly count from one to five until the task period begins. During the 60-second VFT task period, participants will be instructed to generate as many phrases as possible using simple words such as "white," "north," and "big." After completing the phrase constructions, participants will be instructed to resume counting from one to five repeatedly for the duration of the 70-second post-task period. Oxyhaemoglobin, deoxyhaemoglobin, and total haemoglobin will be measured to quantify haemodynamic changes. Fifteen light source probes and sixteen light detector probes will be positioned on the bilateral frontotemporal cortex, maintaining a 3.0 cm distance between each light source and detector probe. In accordance with the 10/20 electrode placement system, the centre of the middle probe will be aligned with FPz, while the lower edge of the probe array will extend from Fp1 to Fp2. For data preprocessing and analysis, raw fNIRS data will be processed using the MATLAB-based Homer3 toolkit. This involves converting light intensity to optical density, detecting and removing motion artefacts, applying correlation-based signal-improvement motion correction, filtering the signal, and performing concentration conversion and block averaging. MNI coordinates will then be obtained for each optode and channel, followed by spatial registration in NIRS-SPM to assign anatomical labels.

***Linguistic Analysis***

Acoustic data will be recorded in a quiet room with minimal background noise on the day of the psychological assessment through a WeChat official account platform. Recordings will be obtained using HUAWEI MatePad BAH3-AN10 tablets (Huawei Technologies Co., Ltd, Shenzhen, China) under the supervision of trained investigators. Participants will read a standardised passage entitled ‘Let Life Be Beautiful Like Summer Flowers’ for approximately three minutes at a normal speaking pace, with the microphone positioned approximately 20 cm from the participant’s mouth. Non-speech segments will be identified and removed using a prespecified voice-activity-detection algorithm. The tablet recording settings are 48 kHz/128 kbps; however, the audio files delivered by the WeChat platform have a sampling rate of 8 kHz and a bit rate of 5.6 kbps. The platform-delivered 8-kHz files will be analysed directly, without subsequent upsampling to 44.1 kHz. Acoustic features will be extracted using openSMILE version 3.0.1 with the emo_large.conf configuration, producing 6,552 features for each recording. Analyses will therefore be limited to the acoustic information retained in the platform-delivered files.

***Facial Expression Analysis***

Facial expression data will be collected using the Muxin application. A HUAWEI MatePad BAH3-AN10 tablet will be securely mounted on a custom-designed stand, with its height and angle adjusted so that each participant's face remains centred in the built-in face-recognition frame. Participants will first read a standardised passage ("Let Life Be Beautiful Like Summer Flowers") to evoke basic emotional expressions. They will then respond spontaneously to open-ended questions (e.g., about self-evaluation or coping strategies for adversity) to capture naturalistic nonverbal behaviour. Video and audio will be recorded using the onboard camera and microphone and saved in MP4 and MP3 formats. For data preprocessing and feature extraction, each video frame will be processed with OpenCV's Haar-feature cascade classifier to detect and crop facial regions. The cropped images will then be analysed with OpenFace 2.0, which produces a comprehensive 709-dimensional geometric feature vector for each frame. These features include gaze-related metrics, head-pose estimates, 2D and 3D facial landmark coordinates, and facial-action-unit intensities. To obtain a fixed-length participant-level representation, each time series of frame-level features will be summarised using six statistical descriptors, mean, variance, skewness, kurtosis, minimum, and maximum, and four multiscale entropy values computed at scales 1–4. This procedure produces 10 summary values for each of the 709 feature time series, resulting in a final 7,090-dimensional temporally pooled facial-behaviour vector for each participant.

***Eye Tracking***

Eye tracking will be observed and recorded using the Shanghai Dikang DEM-2000 eye movement detector (Shanghai Dikang, Shanghai, China). All participants will freely view static images with their left eye. The eye-tracking test will commence by positioning the participant in a semi-reclined position on a calibrated testing chair, ensuring that their line of sight aligns with a display screen 25 cm away at a 33° horizontal angle. The camera detecting corneal reflections of infrared light will be employed to monitor eye movements. An LCD monitor will be utilised to present target images for the eye-tracking tasks. Precise adjustments will be conducted to align the centre of the participant's left eye with the centre of the screen. Subsequently, a calibration procedure will be implemented, guiding the participant to focus on designated points presented on the screen. This will facilitate precise calibration of the eye-tracking system. The calibration process will be repeated until consistent and reliable tracking is attained. A 9-point calibration will be conducted before the initiation of experiments. Based on the reflection of infrared light on the cornea, eye-tracking images will be recorded on the videotape. Three horizontally oriented S-shaped images will be projected directly onto the individual's eyes on a screen 25 cm away. During the initial 15 seconds, S-shaped image 1 will be presented on the screen. Subsequently, two slightly different S-shaped images (2 and 3) will be displayed, each for a duration of 15 seconds. Following these presentations, participants will be asked if they detect any perceived differences between the latter two S-shaped images and the initial S-shaped image. Eye-tracking data will be automatically captured and analysed using a digital eye-tracking recording system. The study will measure 15 eye-tracking features for the three images, including the number of eye fixations (NEF), responsiveness of search scores (RSS), D scores, mean eye scanning length (MESL), and total eye scanning length (TESL).

***Event-Related Potentials — Visual Evoked Potentials (VEPs)***

Visual evoked potentials (VEPs) will be recorded using the Nihon Kohden MEB-9404C evoked potential recorder (Nihon Kohden, Tokyo, Japan). VEP signals will be recorded using gold-cup electrodes, with the active electrode positioned at Oz, the reference electrode at Fpz, and the ground electrode at Pz. The Checkerboard Reversal Pattern will be employed to assess VEPs. To obtain transient responses, checkerboard patterns with 100% contrast will be presented on a television monitor and will undergo contrast reversal at a frequency of two reversals per second. The VEP recordings will be conducted at a distance of 120 cm using the optoelectronic stimulator Galileo Mizar Sirius (EBNeuro, Florence, Italy), at high (15′ checks) and low (55′ checks) spatial frequencies. The VEP waveform exhibits a triphasic waveform, characterised by peaks approximately at 75 ms, 100 ms, and 145 ms following the alteration of the visual stimulus. The peak latencies of the N75, P100, and N145 components will be systematically recorded. Each eye, identified as left (L) and right (R), will undergo two separate tests, denoted as 1 and 2, respectively. Consequently, this procedure will yield a total of 12 distinct VEP features, specifically L1, L2, R1, and R2 for each of the N75, P100, and N145 components.

***Event-Related Potentials — Auditory P300***

Auditory P300 will be recorded using a Dantec Keypoint 4 evoked-potential system (Natus Medical, Denmark) and an active auditory oddball paradigm. Recording electrodes will be placed at Fz and Cz according to the international 10–20 system. M1 and M2 will serve as reference electrodes, and FPz will serve as the ground electrode. Standard and target pure tones will be presented binaurally in a random sequence. Non-target 1,000-Hz tones and target 4,000-Hz tones will be presented at the same intensity of 80 dB HL, with probabilities of 80% and 20%, respectively. Participants will be instructed to press a button when they detect a target tone. A total of at least 300 stimuli will be presented. The recording epoch will include a 100-ms pre-stimulus baseline and a 900-ms post-stimulus interval. Trials with excessive artefact will be excluded according to the prespecified acquisition standard operating procedure. N100, P200, N200, and P300 peak amplitudes and latencies will be extracted at Fz and Cz, yielding 16 prespecified ERP features.

***Heart Rate Variability (HRV)***

Heart rate variability (HRV) measurements (Medeia Medical Technology Co., Ltd, Canada) will be conducted in the Department of Neuroelectrophysiology, the Second Affiliated Hospital of Henan Medical University. This device supports dual-channel physiological data collection, including photoplethysmography (PPG) and electrocardiography (ECG), and permits the adjustment of measurement duration according to specific testing requirements. During the measurement process, spot electrodes will be placed on each participant's chest for ECG recording. A finger-clip sensor will be attached to the index finger of one hand, positioned downward and resting above the thigh, to facilitate PPG measurement. Participants will be seated comfortably in a semi-reclined position and instructed to relax. They will be asked to refrain from speaking or moving during the 5-minute resting HRV data collection. HRV acquisition will use the prespecified 5-minute resting recording described above. Before any outcome-informed analysis, a dated and version-controlled HRV computational specification will define the analysis channel, software and version, inter-beat-interval detection procedure, signal-quality criteria, artefact and ectopic-beat handling, valid-recording and valid-interval requirements, excluded-recording criteria, complete feature vector, feature-computation settings, transformations, and standardisation procedures. No HRV feature selection, outcome association analysis, predictive-model fitting, threshold selection, or performance evaluation will be undertaken before this specification is locked. The specification will be locked before any HRV feature is linked to a questionnaire-defined outcome, trial outcome, or model-performance evaluation. No observed association with an outcome, model-performance result, or threshold-performance result will be used to select or modify the primary feature set, quality-control thresholds, preprocessing parameters, or feature-computation procedure. Any post-lock modification will be documented in a dated version-controlled amendment and reported as exploratory; it will not replace the prespecified primary analysis.

**3. Planned Biological Measurements**

The biospecimen and multi-omics component is exploratory and has not yet been activated. Blood, urine, stool, and saliva are candidate biological matrices, but the matrix-specific collection and processing procedures have not yet been finalised. Before collection of the first sample of a given matrix, a standard operating procedure will specify participant preparation, collection timing, collection materials, specimen volume, processing intervals, centrifugation and aliquoting procedures where applicable, transport conditions, storage temperature, permitted freeze–thaw cycles, and specimen-level acceptance and exclusion criteria. Before any molecular assay is initiated, a separate layer-specific protocol will specify the eligible specimens, assay platform, laboratory procedures, allocation of samples across batches, technical replicates, negative and pooled quality-control samples where applicable, assay-failure criteria, preprocessing, normalisation, and statistical analysis. Appropriate terminology will be used for each resulting data type, including gene expression for transcriptomic data, protein abundance for proteomic data, and metabolite levels for metabolomic data. No biospecimen collection or omics assay under this component will begin until the relevant procedures have been finalised, documented in a dated and version-controlled protocol amendment, and submitted for ethics review where required. The final procedures and the numbers of collected, processed, assay-eligible, and quality-control-eligible specimens will be reported separately for each matrix and omics layer.

Governance of biospecimens and potentially identifiable data. Biospecimens and associated molecular data will be assigned coded study identifiers, with direct identifiers and linkage keys stored separately under restricted access. Access to biospecimens, genomic data, raw voice recordings, facial-expression videos, imaging data, and other sensitive participant-level information will be limited to authorised personnel and governed by the approved consent, ethics provisions, institutional policies, and data-use agreements. Such data will not be deposited in unrestricted public repositories. Derived features, model embeddings, and trained model artefacts will undergo disclosure-risk review before sharing. Biospecimen and data retention, secondary use, and destruction will follow the approved consent materials, protocol amendments, and applicable institutional requirements.

**4. Psychological Interventions**

Group-delivered interventions will generally be conducted with 6–10 participants per group, with a planned mean of eight. Each intervention will be delivered by at least two trained providers at each active site where feasible. Provider and intervention-group identifiers, attendance, intervention exposure, protocol deviations, and fidelity assessments will be recorded for every session. These variables will be used to describe treatment delivery and to account for intervention-group or provider clustering in the statistical analysis.

***Cognitive Behavioural Therapy (CBT)***

Cognitive behavioural therapy (CBT) consists of psychoeducation, behavioural activation, cognitive therapy, and relapse prevention. CBT will be delivered through structured, modular programmes focusing on recognising maladaptive thought patterns, cognitive restructuring, and behavioural change strategies. The provisional dose is 16 sessions over 8 weeks. Online or face-to-face and individual or group delivery remain potential formats within this single CBT trial. The final delivery format, provider-support model, allocation rule, and dated intervention-manual version will be locked before trial activation and the first participant randomisation.

***Interpersonal and Social Rhythm Therapy (IPSRT)***

Based on the principles of social support, social norms, social connection, interpersonal psychotherapy, and psychoeducation, interpersonal and social rhythm therapy (IPSRT) is a psychosocial intervention designed to address circadian rhythm disturbances in university students. IPSRT emphasises stabilising daily biological rhythms (sleep–wake cycles) and managing interpersonal stressors effectively. Sessions aim to improve emotional regulation and decrease the likelihood of symptom recurrence through structured routine tracking. IPSRT will be delivered according to a manualised protocol. The provisional dose is eight weekly sessions over 8 weeks. Online or face-to-face and individual or group delivery remain potential formats; the final format, provider arrangements, and manual version will be locked before trial activation and reconciled with the registered protocol.

***Physical Exercise***

The physical-exercise intervention includes structured aerobic and anaerobic exercise programmes. Group-based formats will be used to enhance adherence, with intensity and duration consistent across participants. The intervention method used in the individual exercise group will combine running and strength training. Aerobic exercise will be guided by the Muxin application, and the distance of each run will be 2 km. The anaerobic exercise will consist of a light warm-up, plank, bridge, bird dog, dead bug, and side plank. Strength training will include planks, sit-ups, push-ups, and elastic-band exercises. After participants complete the running portion alone, they will return to the designated location for strength training. The overall duration will be approximately 45 minutes, including 5 minutes of relaxation. The physical-exercise groups will receive interventions twice a week for a total of six weeks.

***Personalised Music Intervention***

The planned personalised music intervention will be delivered provisionally in a group format and will combine receptive listening and active musical activities. The session content, personalisation procedure, provider requirements, setting, dose, and intervention manual will be finalised before trial activation and the first participant randomisation. No AI system will be incorporated into this trial unless a separate version-locked AI intervention specification, trial-specific protocol, ethics approval, and updated registry record have been completed before recruitment.

***Biofeedback Intervention***

The biofeedback intervention involves group-based training in physiological self-regulation. The experimental instrument selected for this study is the group wireless biofeedback instrument (Free Mind-S/Free Mind-G; Nanjing VISHEE Medical Technology Co., Ltd, Nanjing, China). The system consists of self-balanced training software loaded on a computer and a blood-volume-pulse sensor (also known as a PPG sensor). Participants will use real-time monitoring devices to view physiological markers and use physiological feedback to practise autonomic self-regulation and relaxation techniques. The session frequency, session duration, total number of sessions, programme duration, and home-practice requirements have not yet been finalised. These elements will be specified in the intervention manual and prospectively documented before trial activation and the first participant randomisation.

***Virtual Reality (VR) Therapy***

The planned VR intervention will use immersive 360-degree videos within structured modules for behavioural activation, psychoeducation, and skills practice for emotion management. B6 will be delivered individually, face to face, in a supervised setting using a VR headset. The provisional programme comprises 12 sessions over 4 consecutive weeks, with each session lasting approximately 45 minutes.

The 37-video stimulus library has not yet been finalised. Before recruitment and the first participant randomisation, the final stimulus set will be frozen in a dated, version-controlled B6 stimulus manifest. Each item in the manifest will include a stable identifier, title, source and platform URL, source or channel identifier, duration, verified resolution, access date, library version date, category, selection criterion, content-review decision, and licensing or access status. Videos will be selected from content meeting the prespecified image-quality requirement of at least 4K resolution and will be assigned to one or more of the following categories: animals; sports, dance or arts; adrenaline; travel; and hiking or outdoors. Where permitted, an access-controlled archival copy or file-integrity record will be retained.

At baseline, participants will rank the five content categories. At each session, the facilitator will offer only the version-locked content options specified in the intervention manual for the relevant session and the participant’s highest-ranked available category. No internet search, new download, or unplanned substitution will be permitted after trial activation. If a planned video cannot be used because of technical failure, participant distress, or another documented safety-related reason, the facilitator may use only the pre-authorised replacement specified in the version-locked stimulus manifest. The video identifier, exposure duration, completed module, and reason for any non-completion or replacement will be recorded for every session.

***Expressive Art Intervention***

The expressive-arts intervention will use structured art-making activities, including painting, drawing, clay work, and collage, followed by therapist-guided reflection and emotion-regulation practice. It is intended to support emotional awareness and regulation. Its effects on the trial-specific primary and secondary outcomes will be evaluated in B7. It is based on the belief that the creative process involved in artistic self-expression helps participants resolve emotional conflict, develop interpersonal skills, manage behaviour, reduce stress, increase self-esteem, enhance self-awareness, and achieve personal insight. Each art-therapy session will encourage students to engage their creative side and will employ consistent, standardised art-therapy practices aimed at engaging the mind, body, and spirit of the participant. Each participant will be allowed full freedom of materials to work with, including supplies for painting, drawing, clay work, and collage construction. Sessions will conclude with an art-therapist-guided patient reflection on their own art. Each session will last 60 minutes, including 5 minutes of mindful relaxation and a 15-minute break. The expressive art intervention will be performed three times a week for four weeks, for a total of 12 sessions.

***Five-Sensory Intervention***

The five-sensory stress-regulation intervention will combine structured visual, auditory, olfactory, tactile, and gustatory activities. It is intended to support stress regulation and emotional awareness. The provisional programme comprises 12 sessions over six weeks; the session duration, facilitator procedures, and fidelity criteria will be finalised before trial activation. Its effects on PSS-10 and the prespecified secondary outcomes will be evaluated in B8.

**Supplementary Tables**

**Supplementary Table 1.** Prespecified reference outcomes, labels, and evaluation strategy for the AI tasks of the CAMPUS programme.

| **AI task** | **Reference outcome (target)** | **Index time / prediction horizon** | **Eligible population** | **Positive label** | **Negative label** | **Borderline handling** | **Primary performance measure** | **Diagnostic reference standard** |
| --- | --- | --- | --- | --- | --- | --- | --- | --- |
| **T1. Screening for depressive symptoms (C1)** | PHQ-9 ≥ 10 at the same wave | Index: same assessment wave / Horizon: concurrent | Cohort participants with the questionnaire-defined reference outcome and the predictors required for the relevant prespecified modality-availability block at a given wave | PHQ-9 ≥ 10 | PHQ-9 < 10 | Retained; sensitivity at ≥ 8 and ≥ 12; continuous PHQ-9 as separate regression task | AUROC; AUPRC where outcome imbalance warrants; sensitivity, specificity, positive predictive value, negative predictive value, and F1 score at the locked threshold; calibration plot, calibration intercept and slope, and Brier score | None (questionnaire-defined) |
| **T2. Anxiety symptom screening (C1)** | GAD-7 ≥ 10 at the same wave | Concurrent | As T1 | GAD-7 ≥ 10 | GAD-7 < 10 | Retained; continuous GAD-7 as separate regression task | AUROC; AUPRC where outcome imbalance warrants; sensitivity, specificity, positive predictive value, negative predictive value, and F1 score at the locked threshold; calibration plot, calibration intercept and slope, and Brier score | None (questionnaire-defined) |
| **T3. Insomnia symptom screening (C1)** | ISI ≥ 15 at the same wave | Concurrent | As T1 | ISI ≥ 15 | ISI < 15 | Retained; continuous ISI as separate regression task | AUROC; AUPRC where outcome imbalance warrants; sensitivity, specificity, positive predictive value, negative predictive value, and F1 score at the locked threshold; calibration plot, calibration intercept and slope, and Brier score | None (questionnaire-defined) |
| **T4. Questionnaire-defined BSI-CV positive-screen classification (C1)** | Any BSI-CV item > 0 at the same wave | Concurrent | As T1 | Any BSI-CV item > 0 | All BSI-CV items = 0 | All positive responses trigger the safety protocol (Section 2.3.2) | AUPRC as the principal discrimination measure; AUROC; sensitivity, specificity, positive predictive value, negative predictive value, and F1 score at the locked threshold; calibration plot, calibration intercept and slope, and Brier score | None; clinical assessment follows the safety protocol, not a modelling reference |
| **T5. Incident depressive symptoms (A2)** | Time to the first follow-up PHQ-9 score ≥10; participants lost to follow-up before the prespecified horizon without an observed event will be right-censored. | Index: baseline / Horizon: first subsequent PHQ-9 ≥10 event within five years | Participants with baseline PHQ-9 <10 and the baseline predictors required for the relevant prespecified modality-availability block. | First PHQ-9 ≥ 10 during follow-up | No PHQ-9 ≥10 through the prespecified prediction horizon among participants observed through that horizon. | Participants with scores close to the threshold will be retained and classified according to the prespecified PHQ-9 ≥10 rule; sensitivity analyses will use thresholds of ≥8 and ≥12 and continuous PHQ-9 scores. | Time-dependent discrimination, calibration, and Brier scores at prespecified prediction horizons using censoring-aware methods | None (questionnaire-defined) |
| **T6. Prediction of trial-specific treatment outcomes (C2)** | Continuous change from baseline in the trial-specific primary outcome measured within 7 days after the scheduled final intervention session | Index: prespecified baseline and early-treatment predictors collected before the primary outcome assessment | Trial participants with the required early-phase predictors and post-intervention primary-outcome data | Not applicable to the primary continuous-outcome analysis. | Not applicable to the primary continuous-outcome analysis. | The continuous outcome will be retained without dichotomisation in the primary analysis. No MCID-based responder classification is prespecified for PSS-10 outcomes in B5 or B8. | For the primary continuous-outcome analysis: mean absolute error, root mean squared error, R², and calibration plots. For supplementary responder models: AUROC, AUPRC, sensitivity, specificity, positive predictive value, negative predictive value, F1 score, and calibration | None; all outcomes are questionnaire-defined and do not constitute clinical diagnoses |

Note. AUPRC, area under the precision-recall curve; AUROC, area under the receiver operating characteristic curve; BSI-CV, Chinese version of the Beck Scale for Suicide Ideation; GAD-7, Generalised Anxiety Disorder-7; ISI, Insomnia Severity Index; MCID, minimal clinically important difference; PHQ-9, Patient Health Questionnaire-9. For every task, the questionnaire defining the reference outcome, its individual items, and variables directly derived from it are excluded from the predictor set. No structured diagnostic interview serves as a modelling reference standard; model outputs are screening and risk-stratification results only. Unless otherwise specified, principal performance estimates will be accompanied by participant-level bootstrap 95% confidence intervals. Threshold-specific measures will be calculated at predicted-probability thresholds selected using development data and locked before held-out evaluation. Centre-specific and prespecified subgroup performance will be reported where sample sizes and outcome-event counts permit stable estimation.

**Supplementary Table 2.** Prespecified computational architecture of the multimodal screening and prediction framework.

| **Modality** | **Model input** | **Preprocessing and feature extraction** | **Primary encoder** | **Latent representation** |
| --- | --- | --- | --- | --- |
| **Questionnaire measures** | Eligible scale and subscale scores, excluding the instrument used to define the corresponding concurrent reference outcome | Scoring according to instrument manuals; range and consistency checks; standardisation using development-data parameters | Multilayer perceptron (MLP) applied to the structured score vector | Fixed-dimensional questionnaire latent representation |
| **Sociodemographic and other structured variables** | Age, sex, academic stage, lifestyle factors, medical history, and other eligible structured variables | Encoding of categorical variables and standardisation of continuous variables using development data | Embedding layer for categorical variables followed by an MLP | Fixed-dimensional structured-data latent representation |
| **Voice and acoustic data** | Audio recording of the standardised reading passage | Voice-activity detection and removal of non-speech segments; analysis of the native 8-kHz platform output without subsequent upsampling; extraction of the 6,552-dimensional emo_large.conf feature vector using openSMILE version 3.0.1 | MLP applied to the standardised acoustic feature vector | Fixed-dimensional acoustic latent representation |
| **Language data** | Transcripts of open-ended responses, where available | Transcription quality control; tokenisation; truncation or padding performed within the development pipeline | Pretrained Transformer language encoder used for text only | Fixed-dimensional textual latent representation |
| **Facial-expression data** | Video recordings obtained during the standardised and open-ended tasks | Face detection and cropping with OpenCV; extraction of 709 frame-level OpenFace 2.0 features; temporal pooling using six statistical descriptors and multiscale entropy at scales 1–4, producing a 7,090-dimensional participant-level vector | MLP applied to the temporally pooled facial-feature vector | Fixed-dimensional facial-behaviour latent representation |
| **Eye tracking** | Fixation, scan-path, search, and related eye-movement measures | Quality checks and standardisation of the prespecified eye-tracking feature vector | MLP | Fixed-dimensional eye-tracking latent representation |
| **EEG** | Resting-state spectral and functional-connectivity features | Fixed 20–170-second analysis window; 0.5–45 Hz filtering; prespecified channel-level and recording-level quality control; temporary removal of bad channels; linked-mastoid re-referencing; Extended Infomax ICA with ICLabel-based removal of Eye and Muscle components; post-ICA spherical-spline interpolation; spectral-power and weighted phase-lag-index connectivity features. | MLP applied to the prespecified spectral and connectivity feature vector | Fixed-dimensional EEG latent representation |
| **ERP** | Visual-evoked-potential and auditory-P300 latency and amplitude features | Recording-level quality checks followed by standardisation of the prespecified ERP features | MLP | Fixed-dimensional ERP latent representation |
| **HRV** | Five-minute resting ECG/PPG recording; final HRV feature vector to be defined in a dated, version-controlled computational specification before outcome-informed analysis. | Signal-quality assessment, inter-beat-interval detection, artefact and ectopic-beat handling, valid-recording criteria, feature computation, transformation, and standardisation, all prespecified in the locked HRV computational specification. | MLP | Fixed-dimensional autonomic-function latent representation |
| **fNIRS** | Channel-level haemodynamic responses during the verbal-fluency task | Homer3 processing, including optical-density conversion, motion correction, filtering, concentration conversion, and block averaging; NIRS-SPM spatial registration | MLP applied to prespecified channel-level haemodynamic summary features | Fixed-dimensional haemodynamic latent representation |
| **MRI** | Regional structural and resting-state functional-connectivity features derived from T1-weighted, T2-weighted, and resting-state MRI; the final feature vector will be defined in the locked MRI computational specification. | Before outcome-informed analysis, a dated, version-controlled MRI computational specification will lock the software and version, structural and resting-state preprocessing workflows, template space, atlas/parcellation, quality-control criteria, motion handling, nuisance regression, temporal filtering, connectivity definition, harmonisation procedure, regional feature derivation, and standardisation. | MLP applied to the prespecified regional imaging-feature vector | Fixed-dimensional imaging latent representation |

**Note.** Architecture and task-specific prediction heads. Each modality-specific encoder will produce a fixed-dimensional latent representation. Representations available within a prespecified modality-availability block will be projected to a common dimension and treated as modality tokens. Multi-head self-attention across modality tokens will be followed by a fusion multilayer perceptron and task-specific prediction heads: sigmoid heads for T1–T4, a discrete-time survival head for T5, and a linear head for the primary continuous T6 outcome. Additional sigmoid heads may be used for prespecified exploratory T6 responder analyses, and linear heads for auxiliary continuous-score prediction tasks.

Model development, comparison, and missing modalities. At each resampling stage, all data-dependent preprocessing, fitted feature extraction, encoder fitting, feature selection, class-imbalance handling, and hyperparameter optimisation will be performed within the corresponding training partition only. Hyperparameters, including the common latent dimension, will be selected within the inner participant-grouped folds; outer folds will be used only for performance estimation. Prespecified comparators will include regularised regression and gradient-boosting models fitted to concatenated preprocessed features, together with unimodal models. Models will be developed and evaluated within prespecified modality-availability blocks. Modalities not collected by design will not be imputed or synthetically reconstructed. Incidental missing values within an otherwise collected modality may be handled using procedures fitted exclusively to the relevant training partition. Modality-availability indicators and attention masks may be used only in models prespecified to accommodate variable availability within a block. Transportability within the participating universities will also be examined using the temporal and leave-one-site-out procedures described in Section 2.6.2. Multi-omics data will not be included in the T1–T6 fusion framework and will be analysed separately under Objective C3 in Section 2.6.4. **Abbreviations**: EEG, electroencephalography; ERP, event-related potential; fNIRS, functional near-infrared spectroscopy; HRV, heart-rate variability; MLP, multilayer perceptron; MRI, magnetic resonance imaging; SHAP, SHapley Additive exPlanations.

**Supplementary Table 3.** Planned design features of the eight embedded randomised intervention trials within the CAMPUS master programme protocol.

| **Trial** | **Eligibility criteria** | **Comparator** | **Outcomes** | **Assessment schedule** | **Planned sample size** | **Dose, delivery and provider** | **Randomisation and masking** | **Clustering, adherence, fidelity and analysis** |
| --- | --- | --- | --- | --- | --- | --- | --- | --- |
| B1. Cognitive behavioural therapy (CBT) | mild-to-moderate depressive symptoms (PHQ-9 5–14); no current psychological treatment; no trial-specific contraindication | Trial-specific comparator to be finalised and prospectively specified before trial activation and the first participant randomisation; the comparator and its rationale will be documented in the trial registration, intervention manual, statistical analysis plan, and full trial-specific protocol | Primary: between-group difference in PHQ-9 change from baseline to post-intervention. Secondary: GAD-7, ISI, PSS-10 and BSI-CV; maintenance outcomes at 3 months. | Baseline; within 7 days after the scheduled final intervention session (primary time point); 3-month follow-up | 420 participants (210 per arm); approximately 168 evaluable participants per arm after 20% loss of primary-outcome data | 16 sessions over 8 weeks; online or face-to-face; individual or group format; licensed or appropriately trained therapists | Individual 1:1 randomisation; computer-generated sequence prepared by an independent statistician; central allocation concealment independent of the AI platform; participants and providers not blinded; standardised electronic collection of self-report outcomes; researcher-administered outcome assessors and the primary statistical analyst blinded where feasible | Group format generally involving 6–10 participants, with a planned mean of 8; group and provider identifiers, attendance, intervention exposure, and protocol deviations recorded; manualised protocol, provider training, supervision, and session-level fidelity checks; ITT partially nested mixed-effects analysis with site adjustment and group or provider random effects where applicable; intervention discontinuation distinguished from withdrawal from outcome follow-up; participants discontinuing the intervention encouraged to complete scheduled assessments; missing outcomes addressed using maximum-likelihood mixed-effects models and prespecified sensitivity analyses |
| B2. Interpersonal and social rhythm therapy (IPSRT) | Subclinical insomnia or circadian-rhythm complaints (ISI 8–14); no current psychological treatment; no trial-specific contraindication | Trial-specific comparator to be finalised and prospectively specified before trial activation and the first participant randomisation; the comparator and its rationale will be documented in the trial registration, intervention manual, statistical analysis plan, and full trial-specific protocol | Primary: between-group difference in ISI change from baseline to post-intervention. Secondary: PHQ-9, GAD-7, PSS-10 and BSI-CV; maintenance outcomes at 3 months. | Baseline; within 7 days after the scheduled final intervention session (primary time point); 3-month follow-up | 420 participants (210 per arm); approximately 168 evaluable participants per arm after 20% loss of primary-outcome data | Eight weekly sessions over 8 weeks; online or face-to-face; individual or group format; delivered by trained therapists. | As B1 | As B1 |
| B3. Physical-exercise intervention | mild-to-moderate depressive symptoms (PHQ-9 5–14); able to undertake the planned exercise safely after prespecified screening | Trial-specific comparator to be finalised and prospectively specified before trial activation and the first participant randomisation; the comparator and its rationale will be documented in the trial registration, intervention manual, statistical analysis plan, and full trial-specific protocol | Primary: between-group difference in PHQ-9 change from baseline to post-intervention. Secondary: GAD-7, ISI, PSS-10 and BSI-CV; maintenance outcomes at 3 months. | Baseline; within 7 days after the scheduled final intervention session (primary time point); 3-month follow-up | 420 participants (210 per arm); approximately 168 evaluable participants per arm after 20% loss of primary-outcome data | Two approximately 45-minute sessions per week for 6 weeks; running and strength training; exercise staff with Muxin application guidance | As B1 | Shared exercise-session and provider identifiers recorded; otherwise as B1 |
| B4. Planned personalised music intervention | Subclinical anxiety symptoms (GAD-7 5–14); no current psychological treatment; no trial-specific contraindication | Trial-specific comparator to be finalised and prospectively specified before trial activation and the first participant randomisation; the comparator and its rationale will be documented in the trial registration, intervention manual, statistical analysis plan, and full trial-specific protocol | Primary: between-group difference in GAD-7 change from baseline to post-intervention. Secondary: PHQ-9, ISI, PSS-10 and BSI-CV; maintenance outcomes at 3 months. | Baseline; within 7 days after the scheduled final intervention session (primary time point); 3-month follow-up | 420 participants (210 per arm); approximately 168 evaluable participants per arm after 20% loss of primary-outcome data | Provisional: two group sessions per week for 6 weeks (12 sessions), delivered by trained music therapists or facilitators. The final personalisation procedure, setting, dose, provider requirements, and dated intervention manual will be locked before trial activation. No AI system is included in the current B4 intervention description. | As B1 | As B1 |
| B5. Biofeedback intervention | Moderate perceived stress (PSS-10 14–26); no contraindication to the biofeedback procedure | Trial-specific comparator to be finalised and prospectively specified before trial activation and the first participant randomisation; the comparator and its rationale will be documented in the trial registration, intervention manual, statistical analysis plan, and full trial-specific protocol | Primary: between-group difference in PSS-10 change from baseline to post-intervention. Secondary: PHQ-9, GAD-7, ISI and BSI-CV; maintenance outcomes at 3 months. | Baseline; within 7 days after the scheduled final intervention session (primary time point); 3-month follow-up | 420 participants (210 per arm); approximately 168 evaluable participants per arm after 20% loss of primary-outcome data | Biofeedback programme using Free Mind-S/Free Mind-G devices and trained technicians; weekly frequency, session duration, home-practice requirements and total number of sessions will be finalised in the intervention manual and prespecified before trial activation | As B1 | Device exposure, successful recordings, group/provider IDs and attendance recorded; otherwise as B1 |
| B6. Virtual-reality therapy | mild-to-moderate depressive symptoms (PHQ-9 5–14); no contraindication to VR exposure | Trial-specific comparator to be finalised and prospectively specified before trial activation and the first participant randomisation; the comparator and its rationale will be documented in the trial registration, intervention manual, statistical analysis plan, and full trial-specific protocol | Primary: between-group difference in PHQ-9 change from baseline to post-intervention. Secondary: GAD-7, ISI, PSS-10 and BSI-CV; maintenance outcomes at 3 months. | Baseline; within 7 days after the scheduled final intervention session (primary time point); 3-month follow-up | 420 participants (210 per arm); approximately 168 evaluable participants per arm after 20% loss of primary-outcome data | Twelve face-to-face sessions over 4 weeks; approximately 45 minutes per session; trained VR facilitators | As B1 | VR exposure and adverse effects recorded; shared facilitator effects modelled where applicable; otherwise as B1 |
| B7. Manualised expressive-arts intervention | Subclinical anxiety symptoms (GAD-7 5–14); no current psychological treatment; no trial-specific contraindication | Trial-specific comparator to be finalised and prospectively specified before trial activation and the first participant randomisation; the comparator and its rationale will be documented in the trial registration, intervention manual, statistical analysis plan, and full trial-specific protocol | Primary: between-group difference in GAD-7 change from baseline to post-intervention. Secondary: PHQ-9, ISI, PSS-10 and BSI-CV; maintenance outcomes at 3 months. | Baseline; within 7 days after the scheduled final intervention session (primary time point); 3-month follow-up | 420 participants (210 per arm); approximately 168 evaluable participants per arm after 20% loss of primary-outcome data | Twelve group sessions over 4 weeks (three per week); approximately 60 minutes per session; trained art therapists; activity content, therapist prompts, reflection procedures and fidelity criteria prespecified in the intervention manual | As B1 | As B1 |
| B8. Five-sensory stress-regulation intervention | Moderate perceived stress (PSS-10 14–26); no current psychological treatment; no trial-specific contraindication | Trial-specific comparator to be finalised and prospectively specified before trial activation and the first participant randomisation; the comparator and its rationale will be documented in the trial registration, intervention manual, statistical analysis plan, and full trial-specific protocol | Primary: between-group difference in PSS-10 change from baseline to post-intervention. Secondary: GAD-7, PHQ-9, ISI and BSI-CV; maintenance outcomes at 3 months. | Baseline; within 7 days after the scheduled final intervention session (primary time point); 3-month follow-up | 420 participants (210 per arm); approximately 168 evaluable participants per arm after 20% loss of primary-outcome data | Twelve group sessions over 6 weeks; trained facilitators; visual, auditory, olfactory, tactile and gustatory components, session duration and fidelity criteria prespecified in the intervention manual | As B1 | As B1 |

**Note.** This table describes the planned design framework for the eight embedded trials within the CAMPUS master programme protocol. All trials remain in the planning stage. The information presented here does not constitute a final trial-specific protocol, statistical analysis plan, intervention manual, ethics approval, or registry record. These documents will be finalised before trial activation and the first participant randomisation. CBT, cognitive behavioural therapy; BSI-CV, Chinese version of the Beck Scale for Suicide Ideation; GAD-7, Generalised Anxiety Disorder-7; IPSRT, Interpersonal and Social Rhythm Therapy; ISI, Insomnia Severity Index; ITT, intention-to-treat; PHQ-9, Patient Health Questionnaire-9; PSS-10, 10-item Perceived Stress Scale; VR, virtual reality. All eight trials share the following core design elements: individual-level 1:1 randomisation using computer-generated sequences and central allocation concealment independent of the AI platform; standardised electronic collection of participant-reported outcomes; blinded researcher-administered outcome assessment and blinded primary analysis where feasible; intention-to-treat primary analysis; monitoring of attendance and intervention fidelity using manualised protocols, training, and supervision; and distinction between intervention discontinuation and withdrawal from outcome follow-up. Participants who discontinue the assigned intervention will remain in the intention-to-treat population and will be encouraged to complete all scheduled outcome assessments. Missing outcome data will be handled using maximum-likelihood mixed-effects models based on all available observations, together with prespecified sensitivity analyses. Trial eligibility may be assessed at any cohort screening wave; the AI platform does not determine trial eligibility or randomised allocation. Sensitivity calculations considered intervention-group sizes of 6–10 participants and intraclass correlation coefficients of 0.02–0.10.

**Supplementary Table 4.** Schedule of assessments for the CAMPUS programme.

| **Measure** | **At enrolment/baseline** | **Follow-up schedule** | **Relevant population/subsample** | **Participating sites** | **Planned observations** |
| --- | --- | --- | --- | --- | --- |
| Self-report questionnaires: undergraduate battery | Yes | Every semester (nine follow-up waves) | Full cohort | All seven universities | Approximately 42,000 at baseline (about 6,000 per university); approximately 18,600 expected to remain at the five-year assessment under 15% annual conditional attrition. The mixed-effects analyses use all available waves; the five-year figure is not the number expected to have complete data at all ten waves. |
| Self-report questionnaires: postgraduate battery | Yes | Every semester (nine follow-up waves) | Postgraduate stratum of the full cohort | All seven universities | A subset of the 42,000-participant cohort; postgraduate numbers and completion are reported by site and wave. |
| Voice recording: standardised passage | Yes | Every semester (nine follow-up waves) | Full cohort; platform-based assessment | All seven universities | Approximately 42,000 scheduled at baseline, with subsequent recording opportunities corresponding to retained full-cohort participants at each of the nine semester follow-up waves. Attempted, completed, and quality-control-eligible recordings will be reported separately. |
| Facial-expression recording | Yes | Every semester (nine follow-up waves) | Full cohort; platform-based assessment | All seven universities | Approximately 42,000 scheduled at baseline, with subsequent recording opportunities corresponding to retained full-cohort participants at each of the nine semester follow-up waves. Attempted, completed, and quality-control-eligible recordings will be reported separately. |
| EEG | Yes | Annually while the participant remains under multimodal follow-up (up to four follow-up sessions) | Multimodal subsample; all enrolled participants scheduled for the full core battery | All seven centres using harmonised acquisition and quality-control procedures | Up to 400 new participants per centre per year; up to 2,800 new participants annually and 14,000 unique participants over five annual recruitment cycles. Under staggered recruitment with annual reassessment through programme end, up to approximately 42,000 scheduled modality visits may occur before missed visits, contraindications and quality-control exclusions. |
| ERP: visual evoked potentials and auditory P300 | Yes | Annually while under multimodal follow-up (up to four follow-up sessions) | Multimodal subsample; full core battery | All seven centres | Up to 400 new participants per centre per year; up to 2,800 new participants annually and 14,000 unique participants over five annual recruitment cycles. Under staggered recruitment with annual reassessment through programme end, up to approximately 42,000 scheduled modality visits may occur before missed visits, contraindications and quality-control exclusions. |
| Heart-rate variability | Yes | Annually while under multimodal follow-up (up to four follow-up sessions) | Multimodal subsample; full core battery | All seven centres | Up to 400 new participants per centre per year; up to 2,800 new participants annually and 14,000 unique participants over five annual recruitment cycles. Under staggered recruitment with annual reassessment through programme end, up to approximately 42,000 scheduled modality visits may occur before missed visits, contraindications and quality-control exclusions. |
| Eye tracking | Yes | Annually while under multimodal follow-up (up to four follow-up sessions) | Multimodal subsample; full core battery | All seven centres | Up to 400 new participants per centre per year; up to 2,800 new participants annually and 14,000 unique participants over five annual recruitment cycles. Under staggered recruitment with annual reassessment through programme end, up to approximately 42,000 scheduled modality visits may occur before missed visits, contraindications and quality-control exclusions. |
| Functional near-infrared spectroscopy | Yes | Annually while under multimodal follow-up (up to four follow-up sessions) | Multimodal subsample; full core battery | All seven centres | Up to 400 new participants per centre per year; up to 2,800 new participants annually and 14,000 unique participants over five annual recruitment cycles. Under staggered recruitment with annual reassessment through programme end, up to approximately 42,000 scheduled modality visits may occur before missed visits, contraindications and quality-control exclusions. |
| Magnetic resonance imaging | Yes, unless contraindicated | Annually while under multimodal follow-up (up to four follow-up scans) | Multimodal subsample; all participants without a prespecified MRI contraindication are scheduled for MRI | All seven centres through harmonised 3.0-T scanner procedures and local hospital partnerships | Up to 400 new participants per centre per year; up to 2,800 new participants annually and 14,000 unique participants over five annual recruitment cycles. Under staggered recruitment with annual reassessment through programme end, up to approximately 42,000 scheduled modality visits may occur before missed visits, contraindications and quality-control exclusions. MRI contraindications, incomplete scans and unusable scans are reported separately. |
| Biospecimen collection: blood, urine, stool and saliva | Planned; activation contingent on finalisation and approval of matrix-specific collection and processing SOPs | Follow-up collection schedule to be prespecified separately for each biological matrix before activation | Exploratory biospecimen subsample; eligibility and selection procedures to be prespecified before activation | Participating centres with approved matrix-specific SOPs, trained personnel, and appropriate processing and storage capacity | No fixed evaluable sample size is currently prespecified. Numbers approached, consenting, collected, processed, stored, assayed, and passing quality control will be reported separately for each matrix. |
| Multi-omics profiling | Planned exploratory component; no assay will begin before completion of an assay-specific protocol | Repeat assays according to the prespecified omics question and availability of follow-up specimens | Initial multi-omics subcohort drawn from participants with assay-eligible biospecimens | All seven centres contribute specimens; assays processed in designated laboratories | Initial target of 3,000 participants. Evaluable sample sizes are reported separately for each omics layer after biospecimen loss, assay failure and layer-specific quality control. |

**Note.** EEG, electroencephalography; ERP, event-related potentials; fNIRS, functional near-infrared spectroscopy; HRV, heart rate variability; MRI, magnetic resonance imaging. Questionnaire, voice, and facial-expression assessments are scheduled for the full cohort at baseline and at each of the nine semester follow-up waves. The multimodal subsample is recruited on a rolling basis, with up to 400 new participants per centre per year. Participants enrolled during the first annual recruitment cycle may contribute an enrolment assessment and up to four annual follow-up assessments, whereas those enrolled later will contribute fewer assessments before programme completion. The core modality battery is scheduled for all multimodal participants, subject to participant consent, modality-specific safety contraindications, technical feasibility, and quality-control requirements. The maximum of approximately 42,000 scheduled visits per core modality is a planning estimate under complete attendance and does not represent the expected number of evaluable recordings or scans. Attempted, completed, and quality-control-eligible assessments will be reported separately for each modality. Biospecimen collection and multi-omics profiling are reported separately because not every collected specimen will undergo every omics assay. Non-completion of an individual modality will not affect continued cohort participation or eligibility for the remaining assessments. Reasons for non-completion will be recorded separately as participant refusal, safety contraindication, temporary deferral, technical failure, or quality-control exclusion. Participants will be selected from the eligible core cohort using centre-specific, annual, stratified random invitation by sex and academic stage. Invitation, acceptance, component-specific consent, attendance, contraindication, non-participation, completed modalities, technical failure, and quality-control eligibility will be recorded.

**Supplementary Table 5.** Prespecified multi-omics questions and analysis mapping.

| **Question** | **Eligible population** | **Omics layers** | **Outcome definition** | **Assessment timing** | **Covariates** | **Analysis method** |
| --- | --- | --- | --- | --- | --- | --- |
| **Q1. Molecular correlates of depressive symptom severity** | Multimodal-subsample participants with assay-quality data for the relevant omics layer and the required phenotype and covariate data | Genomic, DNA-methylation, metabolomic and microbiome data where assay-quality data are available; transcriptomic, proteomic and lipidomic data only if generated under prespecified layer-specific protocols | PHQ-9 score (continuous), baseline and repeated | Baseline; follow-up waves | Age, sex, study centre, batch/plate/run, plus layer-relevant covariates (e.g., cell composition, smoking, medication) | Feature-level regression or mixed-effects models; FDR correction within each omics layer |
| **Q2. Molecular predictors of incident depressive symptoms** | Participants with baseline PHQ-9 <10, assay-quality baseline data for the relevant omics layer, and at least one eligible follow-up PHQ-9 assessment | As Q1 (baseline measurements) | First PHQ-9 ≥ 10 at any follow-up wave (Objective A2) | Baseline predictors; semester-wave outcome | As Q1 | Prediction models per Section 2.6.2 (participant-level resampling, held-out data) |
| **Q3. Associations between multi-omics latent factors and depressive-symptom trajectories** | Participants with assay-quality data for the prespecified set of omics layers included in the integration analysis and the required longitudinal symptom data | As Q1 | Estimated PHQ-9 trajectories from mixed-effects models (no omics information used in estimation) | Trajectories estimated from ten waves | As Q1 | MOFA+ latent factors; associations with trajectory parameters |
| **Q4. Pathway enrichment of symptom-associated features** | Not participant-based; analyses use features meeting the prespecified selection rule in Q1 or Q2 | As Q1 | Feature selection rule prespecified per question | After Q1–Q2 primary analyses | Not applicable | KEGG, Reactome, Gene Ontology; identifier mapping, background universe, database release, test and correction prespecified per analysis |
| **Q5. Molecular features associated with trial-specific treatment outcomes** | Trial participants with the required biospecimens, assay-quality data and trial-specific outcome assessments | Available genomic, DNA-methylation, metabolomic and microbiome features; additional transcriptomic, proteomic or lipidomic features only if generated under prespecified layer-specific protocols | Primary: continuous change from baseline on the trial-specific primary scale at the scheduled post-intervention assessment. Exploratory responder outcomes may be defined using reductions of ≥5 points for PHQ-9, ≥4 points for GAD-7 or ≥6 points for ISI. No responder threshold is prespecified for PSS-10 outcomes in B5 or B8. | Baseline biospecimen assessment; post-intervention outcome within 7 days after the scheduled final intervention session; 3-month outcome for secondary analyses | Treatment group, baseline value of the trial-specific primary outcome, site, age, sex, batch/plate/run and other layer-specific technical covariates | Trial-specific regression or mixed-effects models conducted separately for each trial, with continuous change in the trial-specific primary outcome as the principal outcome. Models include treatment group, baseline primary-outcome value, site, demographic covariates and layer-specific technical covariates. Feature × treatment interactions are exploratory and will be examined only where sample size permits. FDR is controlled separately within each trial and omics layer; responder analyses are exploratory. |

**Note.** FDR, false-discovery rate; GAD-7, Generalised Anxiety Disorder-7; ISI, Insomnia Severity Index; MOFA+, Multi-Omics Factor Analysis+; PHQ-9, Patient Health Questionnaire-9; PSS-10, 10-item Perceived Stress Scale. All analyses are exploratory and hypothesis-generating. Omics layers will be analysed only when assay-quality data are available for the relevant question; layers unavailable by design will not be imputed. Quality control, filtering, normalisation, and batch-effect handling will be specified separately for each assay platform before the relevant analysis is undertaken. FDR correction will be applied within each prespecified family of tests defined by research question and omics layer. Internal validation in held-out participants will be performed where sample size permits. Evidence across different omics layers will be described as cross-omics convergence rather than replication, and findings without independent external replication will be reported as discovery findings. No within-participant molecular-change analysis during an embedded trial is prespecified because trial-specific post-intervention biospecimen collection is not currently scheduled.

**Supplementary Table 6. Component-specific safety screening, reasons for deferral or non-completion, and reasonable accommodations.**

| **Study component** | **Prespecified reasons for deferral or non-completion** | **Rationale** | **Reasonable accommodations and continued participation** |
| --- | --- | --- | --- |
| **Core questionnaire assessment** | An acute state preventing the individual from understanding the study information, providing valid informed consent, or completing the core questionnaire assessment despite reasonable accommodation; immediate clinical instability requiring urgent care. | Protects the validity of informed consent and participant safety while avoiding exclusion based on disability alone. | Accessible electronic or large-print formats; screen-reader compatibility; additional time and breaks; verbatim staff administration with the participant's own answers recorded; flexible or divided sessions. Temporary conditions may be reassessed after resolution. |
| **Voice recording** | Acute throat or respiratory symptoms affecting recording; inability or unwillingness to provide a voice sample; inability to obtain a technically usable recording. | These conditions affect feasibility or data validity rather than overall cohort eligibility. | Reschedule after temporary symptoms resolve; provide written instructions and additional preparation time; omit voice recording while retaining all other assessments. |
| **Facial-expression recording** | Participant refusal; inability to obtain an analysable video because of positioning, visual obstruction, or technical failure. | Facial recording is an optional biometric component and is not required for cohort participation. | Accessible positioning; adjustment of camera height and lighting; shorter recording segments; or omission of video recording without loss of cohort eligibility. |
| **EEG and ERP** | Open scalp wounds, active scalp infection, or intolerance or allergy to electrode materials at required placement sites; for visual paradigms involving repetitive visual stimulation, a history of photosensitive seizures or adverse reactions to flickering stimuli. | Electrode application may aggravate affected skin, and visually provocative stimuli may be unsuitable for susceptible participants. | Adjust electrode placement where scientifically acceptable; shorten or divide the session; omit the affected visual task while allowing resting EEG or auditory ERP where safe; complete other modalities. |
| **Heart-rate variability** | Active skin lesions or known intolerance to the required electrodes or sensor materials; inability to maintain the required recording position; acute symptoms making recording inappropriate. | These factors may affect comfort, safety, or signal validity. | Use an alternative compatible sensor placement where permitted by the protocol; use standardised seated rather than supine positioning where appropriate; provide rest breaks or reschedule; omit HRV while retaining other assessments. |
| **Eye tracking** | Acute eye infection or injury; inability to complete calibration; visual or oculomotor impairment preventing valid tracking. | Calibration failure and some ocular conditions affect data validity but are not general cohort exclusions. | Permit compatible corrective lenses; adjust viewing distance, target size, seating, and calibration; allow additional calibration attempts and breaks; omit eye tracking if valid calibration remains impossible. |
| **fNIRS** | Open scalp wounds, active scalp infection, intolerance to cap pressure or optode materials, or inability to obtain adequate optode contact. | Protects skin integrity and identifies circumstances in which a valid optical signal cannot be obtained. | Adjust cap fit and optode position where permitted; carefully separate hair without excluding participants on the basis of hair type; shorten or divide sessions; omit fNIRS while retaining other modalities. |
| **MRI** | An implanted device, foreign body, or other condition that has not been confirmed as MR Safe or MR Conditional under the planned scanning conditions; inability to complete required safety screening; severe claustrophobia or inability to remain safely positioned despite non-sedating accommodations; other contraindications identified by the local MRI safety protocol. | MRI eligibility depends on device-specific conditions and formal local safety screening. | Provide pre-scan orientation, communication throughout scanning, padding and positioning support, and rest breaks where feasible; terminate scanning at the participant's request. Participants who cannot undergo MRI remain eligible for all non-MRI components. |
| **Venous blood collection** | Active infection at the proposed venepuncture site; a bleeding-related condition requiring clinical review; previous severe venepuncture reaction; inability to obtain blood safely. | Minimises avoidable venepuncture-related harm. | Use an experienced phlebotomist; offer supine collection where appropriate and observation after collection; reschedule or omit blood collection without affecting other study participation. |
| **Urine, stool, and saliva collection** | Participant refusal; temporary inability to provide the specimen; collection circumstances that would compromise safety, privacy, or specimen validity. | These are optional biospecimen components and do not determine cohort eligibility. | Provide private and accessible collection facilities, clear accessible instructions, additional time, or home collection where approved; individual specimen types may be omitted. |

**Note.** Component-specific screening will follow the approved protocol, device manufacturer instructions, and applicable site standard operating procedures. A contraindication or inability to complete one component will not constitute exclusion from the overall cohort. Temporary deferrals, participant refusals, safety contraindications, technical failures, and quality-control exclusions will be recorded separately. MRI screening will be performed by qualified local personnel according to the site-specific MRI safety protocol. EEG, electroencephalography; ERP, event-related potential; fNIRS, functional near-infrared spectroscopy; HRV, heart-rate variability; MRI, magnetic resonance imaging.

**Supplementary Table 7. TIDieR-aligned planning matrix for the eight planned embedded interventions.**

| **Trial / intervention** | **Intervention arm and core components** | **Format and setting** | **Provisional dose** | **Planned provider** | **Comparator** | **Planned adherence and fidelity monitoring** | **Manual / version status** |
| --- | --- | --- | --- | --- | --- | --- | --- |
| B1. Cognitive behavioural therapy (CBT) | Manualised CBT comprising psychoeducation, behavioural activation, cognitive restructuring, behavioural change strategies, and relapse prevention. | Online or face-to-face; individual or group delivery. The final delivery format and allocation rule will be locked before trial activation. | Provisional: 16 sessions over 8 weeks. | Licensed or appropriately trained therapists with trial-specific training and supervision. | Trial-specific comparator under development; content, permitted co-interventions, and rationale will be locked before the first participant is randomised. | Attendance, intervention exposure, homework completion where applicable, provider and group identifiers, protocol deviations, and session-level fidelity checklist. | Draft manual under development; a dated version will be locked before trial activation and first randomisation. |
| B2. Interpersonal and social rhythm therapy (IPSRT) | Social-rhythm monitoring, stabilisation of sleep-wake and daily routines, interpersonal strategies, psychoeducation, and relapse-prevention planning. | Online or face-to-face; individual or group delivery. The final format will be reconciled with the registered protocol before recruitment. | Provisional: eight weekly sessions over 8 weeks; delivery format and provider arrangements will be finalised before trial activation. | Therapists trained in the trial-specific IPSRT manual, with supervision. | The trial-specific comparator will be reported exactly as specified in the final registered protocol, including usual-support access and contact frequency. | Attendance, completion of rhythm-monitoring records, intervention exposure, provider and group identifiers, protocol deviations, and session-level fidelity checklist. | Draft manual aligned with the registered protocol; final dated version to be locked before recruitment and randomisation. |
| B3. Structured physical exercise | Structured running and strength-training programme, including warm-up, aerobic activity, core and resistance exercises, and relaxation. | Supervised group sessions with individually completed running components where specified; campus exercise setting with Muxin application guidance. | Provisional: two approximately 45-minute sessions per week for 6 weeks (12 sessions). | Appropriately trained exercise staff or exercise physiologists, with trial-specific safety and protocol training. | Trial-specific comparator under development and to be locked before the first randomisation. | Attendance, completed sessions, exercise exposure, safety events, provider and group identifiers, protocol deviations, and fidelity checklist. | Draft intervention manual under development; final dated version to be locked before trial activation. |
| B4. Planned personalised music intervention | Structured receptive listening and active music activities with a planned personalised music procedure. | Planned group delivery in a standardised campus setting; final delivery procedures will be reconciled with the registered protocol before recruitment. | Provisional: two sessions per week for 6 weeks (12 sessions). | Trained music therapists or facilitators with protocol-specific training and supervision. | The comparator will be reported exactly as specified in the final registered protocol, including access to usual campus support and contact procedures. | Attendance, completed exposure, records of the personalised music activities delivered, provider and group identifiers, protocol deviations, and session-level fidelity checklist. | Draft manual under development; final dated version to be locked before recruitment and randomisation. No AI system is included in the current B4 intervention description. |
| B5. Biofeedback intervention | Group-based physiological self-regulation training using Free Mind-S / Free Mind-G devices and blood-volume-pulse feedback, together with guided relaxation practice. | Planned group delivery in a supervised campus or laboratory setting; home practice remains under consideration. | Not yet finalised; frequency, session duration, total number of sessions, and programme duration will be locked before trial activation. The current programme-level planning range is 8–16 sessions over 4–12 weeks. | Trained technicians or therapists with device-specific and protocol-specific training and supervision. | Trial-specific comparator under development and to be locked before the first randomisation. | Attendance, successful device recordings, duration of exposure, home practice if applicable, provider and group identifiers, adverse events, deviations, and fidelity checklist. | Draft manual under development; dose and final dated version to be locked before trial activation. |
| B6. Virtual-reality therapy | Planned immersive modules for behavioural activation, psychoeducation, and structured skills practice using a 37-video library that will be frozen and version-locked before recruitment and the first participant randomisation. | Individual, face-to-face delivery in a supervised setting using a VR headset. | Provisional: 12 sessions over 4 weeks; approximately 45 minutes per session. | Trained VR facilitators with protocol-specific safety training and supervision. | Trial-specific comparator under development and to be locked before the first randomisation. | Attendance, VR exposure time, video identifier, module completion, adverse effects, facilitator identifier, protocol deviations, permitted substitutions and reasons, and session-level fidelity checklist. | Draft intervention manual and stimulus library under development. Before recruitment and first randomisation, the final 37-video library will be frozen in a dated, version-controlled stimulus manifest; the final manual will specify the session-specific video-selection and replacement procedures. |
| B7. Manualised expressive-arts intervention | Structured art-making activities using painting, drawing, clay, or collage, followed by therapist-guided reflection and emotion-regulation practice. | Group delivery in a standardised campus setting. | Provisional: three approximately 60-minute sessions per week for 4 weeks (12 sessions). | Trained art therapists with trial-specific training and supervision. | Trial-specific comparator under development and to be locked before the first randomisation. | Attendance, completion of core activities, therapist and group identifiers, protocol deviations, and session-level fidelity checklist. | Draft activity and therapist manual under development; final dated version to be locked before activation. |
| B8. Five-sensory stress-regulation intervention | Structured visual, auditory, olfactory, tactile, and gustatory activities intended to support stress regulation and emotional awareness. | Planned group delivery in a standardised campus setting. | Provisional: 12 sessions over 6 weeks; session duration will be locked before activation. | Trained facilitators with protocol-specific training and supervision. | Trial-specific comparator under development and to be locked before the first randomisation. | Attendance, completion of sensory modules, facilitator and group identifiers, adverse reactions where applicable, deviations, and session-level fidelity checklist. | Draft intervention manual under development; session duration and final dated version to be locked before activation. |

**Note.** All eight embedded trials remain in the planning stage and have not commenced recruitment or randomisation. Only B2 and B4 currently have registry entries. The remaining six trials will each be registered prospectively before recruitment and the first participant randomisation. This table summarises provisional intervention components and implementation considerations and is not a final intervention manual. The programme-level planning range is 8-16 sessions over 4-12 weeks; the trial-specific dose shown above is provisional. For every trial, the final intervention format, dose, provider requirements, comparator, adherence and fidelity procedures, and dated manual version will be locked and prospectively documented before trial activation and the first participant randomisation. CBT is one embedded trial; individual/group and online/face-to-face delivery are potential delivery variants rather than separate trials. TIDieR, Template for Intervention Description and Replication; CBT, cognitive behavioural therapy; IPSRT, interpersonal and social rhythm therapy; PPG, photoplethysmography; VR, virtual reality.

**Supplementary Table 8. Chronology of ethics-approved cohort documentation and manuscript-associated CAMPUS protocol versions.**

| **Document** | **Date** | **Document status** | **Relationship to cohort implementation** | **Main content** |
| --- | --- | --- | --- | --- |
| Ethics-approved Henan-site implementation document: “Intelligent Assessment of University Students’ Mental Health and the Effects of Group Psychological Interventions” | 8 April 2025 | Pre-enrolment ethics-approved Henan-site implementation document | Governed initial cohort recruitment and university-student mental-health assessment at Henan Medical University from September 2025 | Henan-site mental-health assessment and initial cohort procedures; did not constitute a multicentre ethics approval or the later CAMPUS master protocol |
| CAMPUS master protocol v1.0 | 10 May 2026 | First manuscript-associated, version-controlled master protocol | Developed after cohort initiation but before any post-enrolment longitudinal-cohort outcome data had been viewed, exported, or analysed; it did not replace the historical fact that enrolment began in September 2025 | Consolidated the multicentre programme, planned trials, multimodal assessments, and analytical framework |
| CAMPUS master protocol v2.0 | 5 September 2026 | Current version | Peer-review revision | Clarified chronology, trial status, analysis plans, reproducibility procedures, and reporting terminology |

**Note.** Before Version 1.0 was dated 10 May 2026, personnel developing the CAMPUS master protocol and the statistical and AI analysis plans had not received, viewed, exported, merged, summarised, or analysed individual-level or aggregate post-enrolment longitudinal-cohort outcome data. No data extracts, descriptive summaries, statistical outputs, or other reports containing post-enrolment cohort outcome information had been provided to those personnel. A site safety pathway for positive suicide-risk screens had been established before implementation; however, no participant generated a positive safety screen requiring case-level review or escalation before 10 May 2026. Procedures and analyses formalised after enrolment are not represented as having been specified before enrolment. Ethics approval for each additional participating university will be obtained separately from the responsible local ethics committee before site activation, participant enrolment, or data collection.

**Supplementary Table 9. Decision-level traceability of key CAMPUS design and analysis specifications.**

| **Key decision** | **Pre-enrolment ethics-approved Henan-site implementation document, 8 April 2025** | **CAMPUS master protocol v1.0, 10 May 2026** | **CAMPUS master protocol v2.0, 5 September 2026** | **Interpretation in the present report** |
| --- | --- | --- | --- | --- |
| Recruitment and sampling approach | Explicitly specified the Henan-site sampling approach. | Consolidated the programme-level multicentre recruitment framework. | Clarified multistage probability sampling, design-based descriptive analyses, and weighting procedures. | The initial Henan-site sampling procedures were specified before enrolment; later versions extended and clarified the multicentre framework. |
| Assessment instruments | Explicitly listed the planned psychological assessment instruments. | Consolidated the assessment framework for the CAMPUS programme. | Clarified instrument terminology, scoring descriptions, and references where required. | Instruments documented in the Henan-site file are distinguished from later programme-level refinements. |
| Longitudinal follow-up arrangement | Explicitly specified the follow-up arrangement. | Consolidated the five-year, semester-based longitudinal schedule for the multicentre programme. | Clarified assessment timing and related reporting details. | The existence of follow-up was specified before enrolment; later versions formalised the programme-level schedule. |
| A1 primary cohort outcome | Explicitly identified PHQ-9 as the primary longitudinal cohort outcome. | Formally defined A1 as the primary longitudinal analysis of depressive-symptom trajectories. | Clarified the estimand, weighting approach, missing-data procedures, and interpretation of secondary analyses. | PHQ-9 was specified before enrolment as the primary cohort outcome; the detailed A1 analytical framework was formalised subsequently. |
| Incident depressive-symptom event | Not formally specified. | First formally defined as the first follow-up PHQ-9 score ≥10 among participants with baseline PHQ-9 <10. | Clarified prediction horizons, censoring, threshold sensitivity analyses, and questionnaire-defined terminology. | This is a later analytical event definition and is not represented as having been prespecified before enrolment. |
| Suicide-safety procedures | Explicitly included the response pathway for positive suicide-risk screens. | Consolidated programme-level safety procedures. | Clarified electronic alerts, qualified professional assessment, referral, emergency escalation, documentation, and safety oversight. | The pre-enrolment safety pathway is distinguished from the later detailed research-programme safety specification. |
| Physiological and neurofunctional assessments | Explicitly included EEG, ERP, HRV, fNIRS, and MRI assessments. | Consolidated the planned multimodal framework for the CAMPUS programme. | Clarified modality availability, quality-control principles, and reproducibility procedures for HRV and MRI feature extraction. | The planned modalities were documented before enrolment; later versions added technical and analytical detail. |
| Statistical and AI analytical framework | Not formally specified. | First formally specified the programme-level statistical and AI analytical framework. | Clarified estimands, weighting, missing-data handling, validation procedures, prediction targets, performance metrics, and reproducibility safeguards. | The detailed statistical and AI framework was formalised after cohort initiation and before post-enrolment cohort outcome-data analysis. |

**Note.** “Explicitly specified” indicates that the relevant procedure or decision was documented in the identified record. “Not formally specified” indicates that the item was not documented in the pre-enrolment ethics-approved Henan-site implementation document. The Henan-site document governed the initial Henan Medical University implementation and did not constitute a multicentre CAMPUS master protocol. Later master-protocol specifications are not represented as having been fixed before cohort enrolment.
